# Integrated Bulk and Single-Cell Transcriptomic Analysis Followed by Clinical Validation Reveal Programmed Cell Death-Related Shared Molecular Signatures in OA and MDD

**DOI:** 10.3390/ijms27125154

**Published:** 2026-06-06

**Authors:** Jihua Liu, Zehao Hu, Zixuan Xu, Tao Xiao, Qiuxuan Huang, Liangji Liu, Zenan Wu

**Affiliations:** 1 School of Clinical Medicine, Jiangxi University of Chinese Medicine, Nanchang 330004, China; jihua_liu@icloud.com (J.L.); p13672261998@163.com (Z.X.); 15779631960@163.com (T.X.); 2 The Second Clinical College, Guangzhou University of Traditional Chinese Medicine, Guangzhou 510000, China; 20231121685@stu.gzucm.edu.cn (Z.H.); huangqiu02@163.com (Q.H.)

**Keywords:** osteoarthritis, major depressive disorder, programmed cell death, comorbidity, bioinformatics, immune infiltration

## Abstract

Osteoarthritis (OA) and major depressive disorder (MDD) share inflammatory and oxidative stress pathways, but the role of programmed cell death (PCD) in their comorbidity remains unclear. This study used independent OA synovial and MDD peripheral blood transcriptomic datasets—not a unified comorbid discovery cohort—to identify candidate PCD-related molecular signatures commonly dysregulated in both conditions. Transcriptomic data from OA synovium and MDD brain tissues were obtained from GEO (six training [three OA synovial and three MDD peripheral-blood], seven validation, and two single-cell RNA-seq datasets). Differentially expressed genes (DEGs) were identified, and PCD-related DEGs were screened. Machine learning (LASSO, SVM-RFE, Random Forest) was used to identify hub PCD-DEGs from the OA training set. WGCNA identified MDD-associated modules for comorbidity-gene selection. Functional enrichment, immune infiltration, scRNA-seq localization, and clinical validation (qRT-PCR/WB) were performed. From the OA cohort, four hub PCD-DEGs (CDKN1A, CX3CR1, INHBB, RHOB) showed moderate diagnostic value for OA (nomogram AUC = 0.82). Eight candidate genes (VAMP8, PDK4, P2RX4, ITM2C, IL10RA, HSP90AA1, CTSO, CRIP1) were commonly dysregulated across both OA and MDD datasets. Immune infiltration revealed upregulated B memory cells, plasma cells, Tregs, and neutrophils in OA, and neutrophils in MDD. scRNA-seq localized CDKN1A/RHOB to OA synovial cells and HSP90AA1/ITM2C to MDD neurons. Enrichment analyses highlighted TNF signaling, apoptosis, and stress responses in both diseases. An independent OA–MDD clinical cohort confirmed differential expression of CDKN1A, RHOB, ITM2C, and HSP90AA1. This study identifies four PCD-related hub genes associated with OA and eight candidate comorbidity genes showing common dysregulation across OA and MDD datasets and in an independent clinical cohort. These findings generate hypotheses about shared inflammatory pathways linking OA and MDD. As these associations derive from independent disease-specific cohorts rather than a true comorbid discovery cohort, they represent candidate signatures requiring functional validation rather than established mechanisms.

## 1. Introduction

Osteoarthritis (OA) and major depressive disorder (MDD) are two common chronic diseases that significantly impact global health [1,2]. OA is characterized by cartilage degeneration, synovitis, subchondral bone remodeling, osteophyte formation, and inflammation of the infrapatellar fat pad [3]. MDD, on the other hand, is primarily characterized by persistent low mood, neuroinflammation, and disruption of neuroendocrine pathways [4,5]. Risk factors for OA include aging, being female, being obese, having a genetic susceptibility, and having had a joint injury [6,7]. MDD, on the other hand, is associated with genetic factors, chronic stress, and systemic inflammation [8]. Epidemiological studies estimate that more than 500 million adults worldwide suffer from OA, while approximately 280 million suffer from MDD [9,10]. Both conditions impose a significant burden on healthcare systems and economies [11,12]. In osteoarthritis, the synovium facilitates the exchange of nutrients and waste products in the cartilage. It forms a functional unit with the infrapatellar fat pad to regulate joint stability and inflammation [13]. However, chronic synovial inflammation in OA drives cartilage matrix degradation, releasing proteoglycans and collagen fragments into the synovial fluid and thereby exacerbating joint degeneration [14,15]. Similarly, chronic neuroinflammation in depression disrupts neuronal homeostasis, leading to synaptic dysfunction and emotional disturbances [16]. Emerging evidence suggests that OA and MDD share common inflammatory and oxidative stress pathways, which may explain their comorbidity [17,18]. Understanding the molecular mechanisms underlying the association between OA and MDD, particularly with regard to their shared inflammatory and immune dysregulation, is crucial for identifying novel biomarkers and therapeutic targets to improve the early diagnosis and management of these comorbid conditions.

Programmed cell death (PCD) is a genetically regulated process critical for homeostasis and disease initiation [19]. Diverse PCD pathways (apoptosis, autophagy, necroptosis, pyroptosis, ferroptosis, cuproptosis) are separately involved in OA and MDD pathogenesis, while the comorbidity of these two disorders has gained increasing attention [20]. In OA, apoptosis triggers chondrocyte loss and cartilage degeneration, autophagy mitigates cellular stress, and necroptosis/pyroptosis exacerbates synovial inflammation. Ferroptosis and cuproptosis, related to oxidative stress and metal ion dysregulation, are associated with neurological diseases; iron/copper overload increases OA and MDD risk, and ferroptosis inhibition ameliorates related lesions [21,22]. Additionally, PCD crosstalk (e.g., PANoptosis) contributes to inflammatory and neurodegenerative diseases, but its role in OA–MDD comorbidity remains underexplored [23,24].

**Figure 1:**
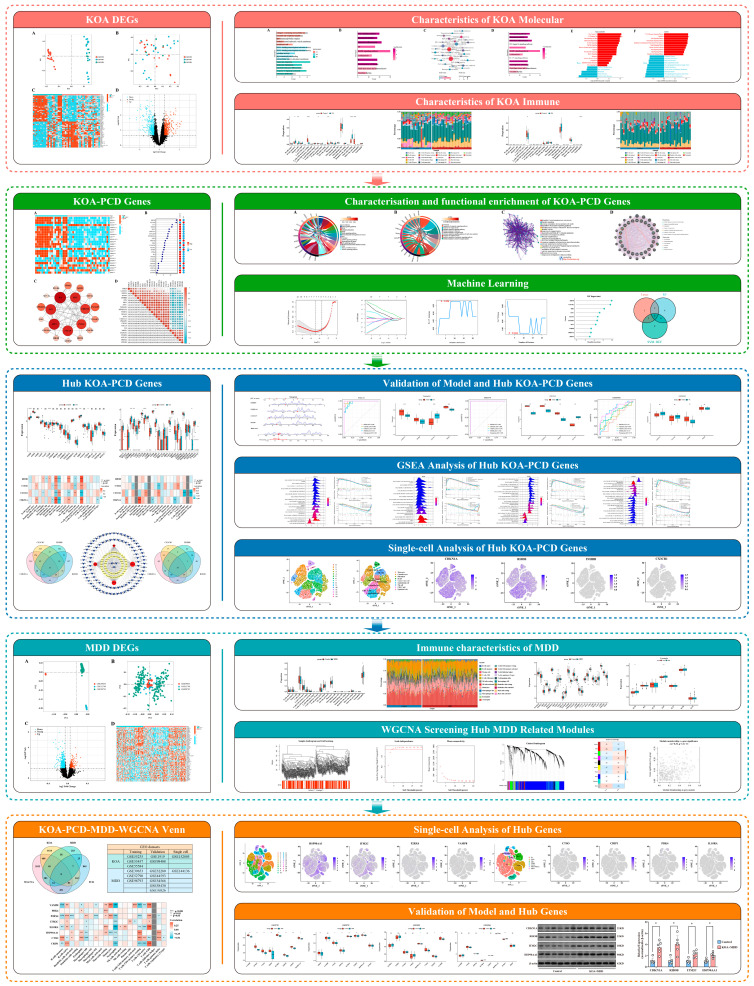
Flow chart for comprehensive analysis of PCD-related genes in OA and MDD. * *p <* 0.05, ** *p <* 0.01, *** *p <* 0.001.

Bioinformatics enables systematic exploration of complex disease mechanisms [25,26]. Although molecular characteristics of OA and MDD have been separately described [27,28], their shared pathways—chronic inflammation, oxidative stress, and immune dysfunction—remain underexplored in the context of comorbidity [17]. This study aims to identify comorbidity-associated genes and elucidate their roles in molecular subtypes, biomarkers, and immune infiltration (Figure 1), providing insights for integrated diagnosis and targeted therapy.

## 2. Results

### 2.1. Data Processing and Differential Genes Screening

**Figure 2:**
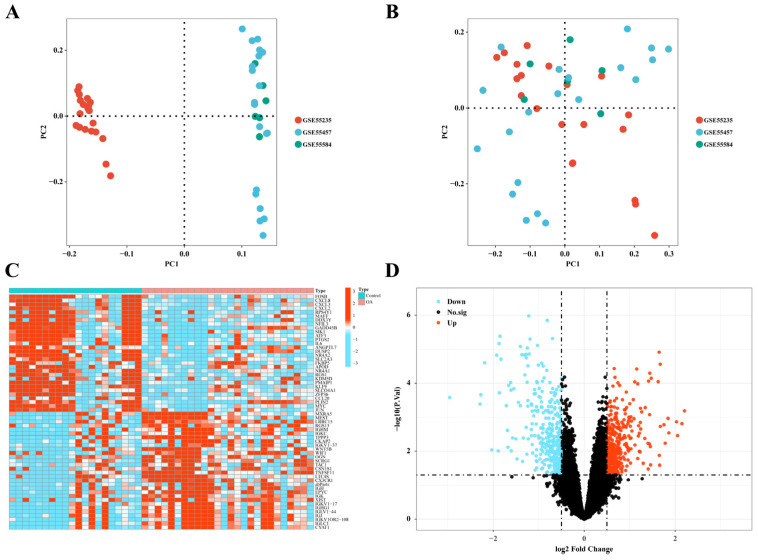
Data processing and DEGs analysis of OA: (**A**,**B**) PCA plots demonstrating sample distribution before and after batch effect correction; (**C**) heatmap of top 60 OA DEGs showing expression patterns in normal synovial vs. OA synovial samples; (**D**) volcano plot of OA DEGs.

To construct the training cohort, three synovial datasets based on the platform GPL96 were integrated, comprising 20 normal and 26 OA synovial samples. After batch effect removal, PCA confirmed effective data integration (Figure 2A,B). Differential expression analysis was performed using the limma R package, identifying 2365 differentially expressed genes (DEGs) in OA, of which 1294 were upregulated, and 1071 were downregulated (Table S3). The top 60 DEGs ranked by significance are presented in a heatmap (Figure 2C), and the overall distribution of DEGs is visualized in a volcano plot (Figure 2D).

### 2.2. Enrichment Analysis of OA

**Figure 3:**
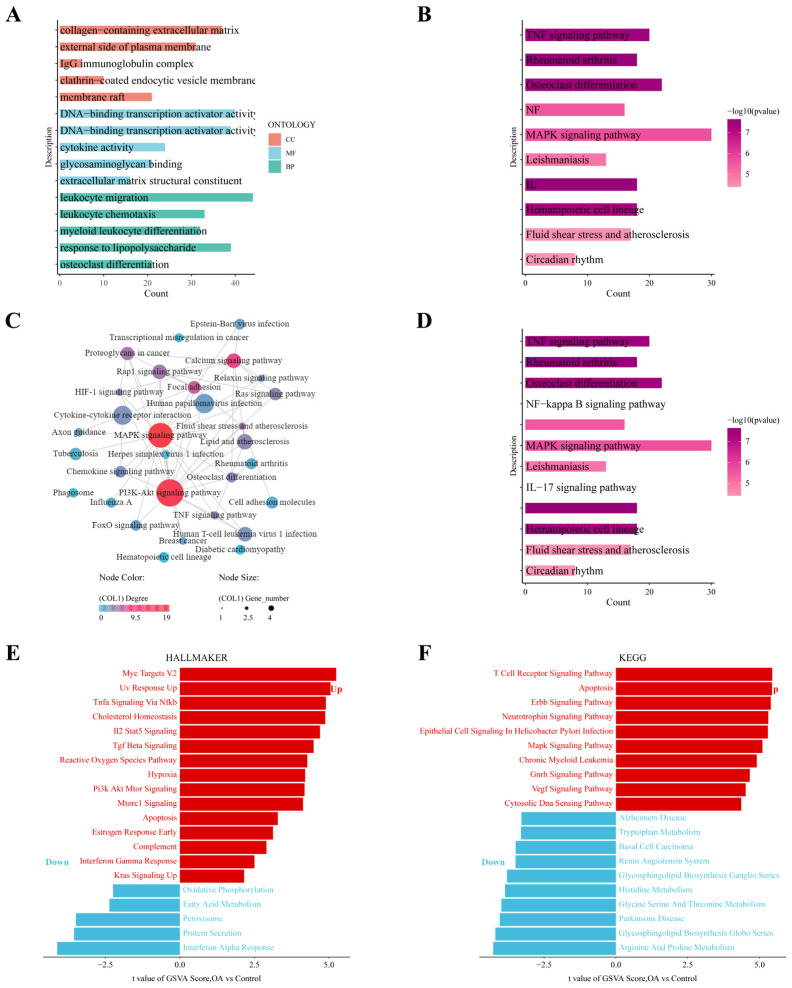
Functional enrichment analysis of DEGs: (**A**) GO enrichment; (**B**) top 10 enriched KEGG pathways; (**C**) KEGG pathway interaction network; (**D**) DO enrichment; (**E**) bar map of GSVA enrichment scoring of HALLMARKS pathways; (**F**) scored bar graph of KEGG pathway GSVA enrichment.

To clarify the functional characteristics of OA-related differentially expressed genes (DEGs), GO, KEGG, DO enrichment, and GSVA analyses were conducted. GO enrichment indicated that DEGs were mainly enriched in collagen-containing extracellular matrix, leukocyte migration, cytokine activity, and other items (Figure 3A). KEGG analysis showed significant enrichment in immune and inflammatory pathways, including TNF, PI3K–Akt, and MAPK signaling pathways (Figure 3B,C). DO enrichment associated DEGs with osteoarthritis, nephritis, atherosclerosis, and other related diseases (Figure 3D). To clarify the functional characteristics of OA-related differentially expressed genes (DEGs), GO and KEGG enrichment analyses were performed separately for upregulated and downregulated genes. For upregulated DEGs, GO enrichment indicated that they were predominantly involved in leukocyte-mediated immunity, positive regulation of leukocyte activation, and immune response-regulating cell surface receptor signaling pathway (Figure S2A). KEGG analysis further revealed significant enrichment in immune-related pathways, including hematopoietic cell lineage and rheumatoid arthritis (Figure S2B). In contrast, downregulated DEGs were mainly enriched in response to hypoxia, fat cell differentiation, and positive regulation of cytokine production (Figure S2C). KEGG pathway analysis showed that these genes were significantly associated with FoxO, TNF, PI3K–Akt, and MAPK signaling pathways, as well as lipid and atherosclerosis (Figure S2D). GSVA based on HALLMARK and KEGG gene sets revealed pathway changes in OA: HALLMARK pathways such as TNFα Signaling via NF-κB were upregulated, while Oxidative Phosphorylation and Fatty Acid Metabolism were downregulated (Figure 3E); KEGG pathways, including apoptosis and T cell receptor signaling, were upregulated, whereas multiple metabolic pathways were downregulated (Figure 3F).

### 2.3. PPI and Correlation Analysis of PCD-DEGs

**Figure 4:**
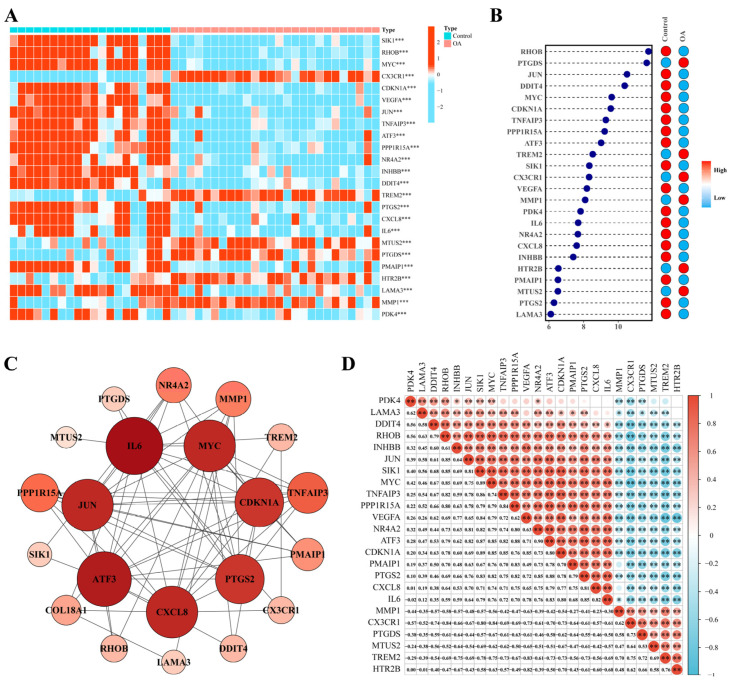
PPI and correlation analysis of PCD-DEGs: (**A**) heatmap of differential expression of PCD-DEGs; (**B**) dot-bar heatmap summarizing the distinct expression trends of PCD-DEGs, providing a quantitative ranking to complement the comprehensive heatmap in panel A; (**C**) PPI analysis of PCD-DEGs; (**D**) correlation heatmap of PCD-DEGs. * *p <* 0.05, ** *p <* 0.01, *** *p <* 0.001.

A total of 24 PCD-related differentially expressed genes (PCD-DEGs) were identified by intersecting DEGs with curated PCD-related genes, including 6 upregulated and 18 downregulated genes in OA (Figure 4A; Table S4). A dot-bar heatmap showed the expression patterns of these PCD-DEGs (Figure 4B). A protein–protein interaction (PPI) network was constructed with Cytoscape, and IL6, JUN, ATF3, CXCL8, PTGS2, CDKN1A, and MYC presented the strongest protein interactions (Figure 4C). Correlation analysis revealed obvious heterogeneity among the 24 PCD-DEGs. CX3CR1, PTGDS, and other genes showed strong negative correlations, while PDK4, RHOB, INHBB, and other genes exhibited strong positive correlations (r = 0.62–0.90 and r = −0.44 to −0.84, respectively) (Figure 4D). A systematic classification of the 24 PCD-DEGs according to curated programmed cell death (PCD) pathway annotations revealed a marked enrichment in apoptosis: 20 genes (83.3%) were assigned to the apoptosis category, whereas only PTGS2 (4.2%) was associated with ferroptosis, two genes (8.3%) with pyroptosis/inflammation (IL6 and CXCL8), and CX3CR1 (14.2%) with multiple PCD modalities. Collectively, these findings indicate that the 24 PCD-DEGs were predominantly enriched in the apoptosis pathway (approximately 83%), rather than being uniformly distributed across all PCD modalities (Figure S2).

### 2.4. Enrichment Analysis of PCD-DEGs

**Figure 5:**
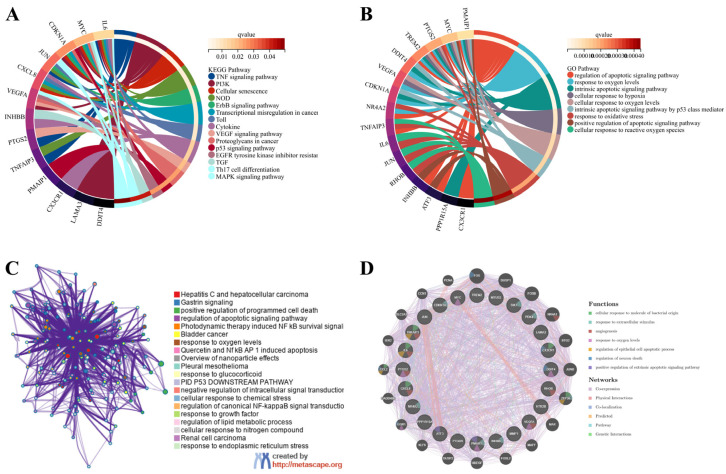
Functional enrichment and network analysis of PCD-DEGs: (**A**) KEGG pathway chord diagram of PCD-DEGs; (**B**) GO term chord diagram of PCD-DEGs; (**C**) GO/KEGG clustered enrichment analysis of PCD-DEGs; (**D**) PPI network of co-expressed genes generated via GeneMANIA.

To explore the functional roles of PCD-DEGs in OA, GO and KEGG enrichment analyses were performed. KEGG analysis revealed enrichment in pathways largely overlapping with those of total DEGs, including TNF, PI3K, and MAPK signaling (Figure 5A). GO analysis showed associations with biological processes such as regulation of apoptotic signaling, response to oxygen levels, p53-mediated intrinsic apoptotic signaling, cellular response to hypoxia and reactive oxygen species, oxidative stress response, positive regulation of apoptotic signaling, gastrin signaling, and photodynamic therapy-induced NF-κB survival signaling (Figure 5B,C). A GeneMANIA network integrating PCD-DEGs with co-expressed partners showed interaction contributions: co-expression (72.87%), physical interactions (17.15%), co-localization (6.57%), genetic interactions (0.46%), predicted interactions (1.78%), and pathway associations (1.17%) (Figure 5D).

### 2.5. Identification of Hub PCD-DEGs

**Figure 6:**
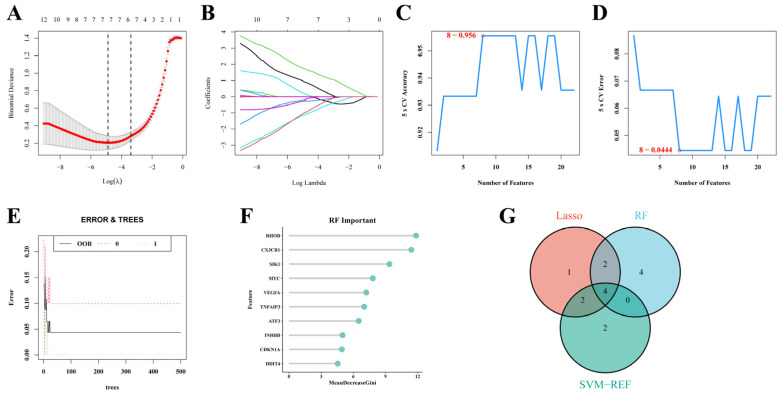
Machine learning-based biomarker screening of Hub PCD-DEGs: (**A**,**B**) LASSO coefficient profiles showing feature selection trajectories, each colored solid line corresponds to a gene in the input data; colors are used solely for visual distinction between genes.; (**C**) cross-validation correlation (r = 0.956) for model robustness; (**D**) SVM-RFE accuracy optimization with 5-fold CV; (**E**) Random Forest error stabilization beyond 10 features; (**F**) RF variable importance ranking; (**G**) integrative Venn diagram of hub genes identified by LASSO, SVM-RFE, and RF algorithms.

Three machine learning algorithms were used to identify hub PCD-DEGs (Table S5). LASSO regression screened seven PCD signature genes (Figure 6A,B). SVM-RFE analysis achieved optimal model performance with 24 features (Figure 6C,D). Random Forest analysis selected ten signature genes (Figure 6E,F). The intersection of the three algorithms ultimately identified four hub PCD-DEGs: CDKN1A, CX3CR1, INHBB, and RHOB (Figure 6G).

### 2.6. Construction of a Diagnostic Model and ROC Curve for OA

**Figure 7:**
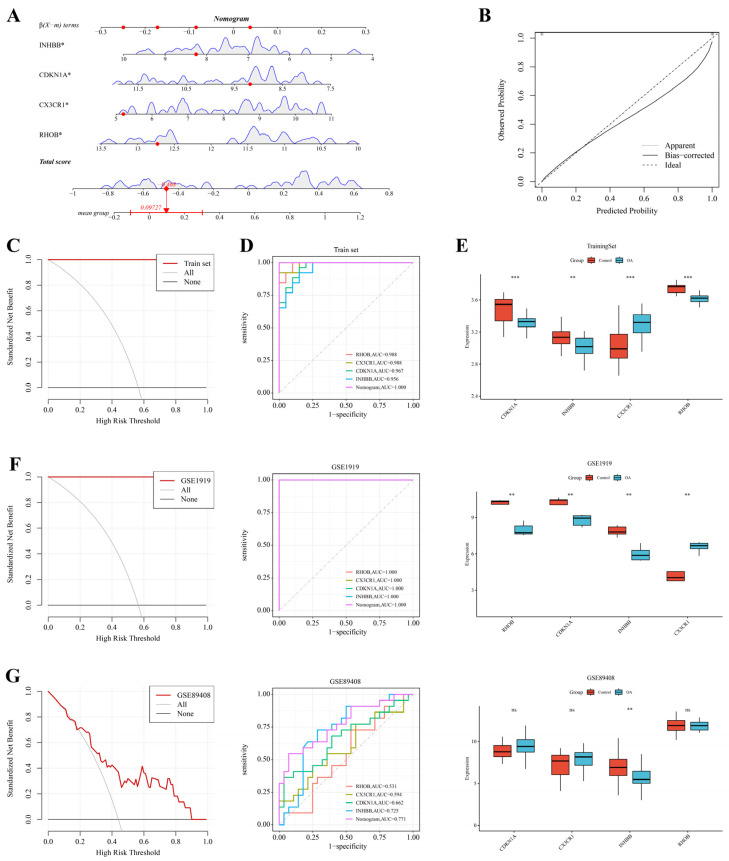
Construction of OA diagnostic model, ROC curves, and validation of Hub PCD-DEGs: (**A**) nomogram for diagnosis of OA; (**B**) calibration curves for assessing the predictive accuracy of the nomogram; (**C**) clinical decision curve of the diagnostic model; (**D**) ROC curve analysis of Hub PCD-DEGs and nomogram in the training set; (**E**) box plots of Hub PCD-DEGs in the training set; (**F**) clinical decision curves, ROC, and box plots analysis of Hub PCD-DEGs and nomogram in the GSE1919 dataset; (**G**) clinical decision curves, ROC, and box plot analysis of Hub PCD-DEGs and nomogram in GSE89408 dataset. * *p <* 0.05, ** *p <* 0.01, *** *p <* 0.001.

A diagnostic model for OA was constructed using the four hub PCD-DEGs (Figure 7A). The calibration curve showed good agreement between predicted and actual risks (Figure 7B), and decision curve analysis indicated favorable net benefit across threshold probabilities (Figure 7C), robust discriminative performance. In the training set, all four individual genes and the nomogram achieved AUC > 0.9, indicating high diagnostic value (Figure 7D,E). The nomogram achieved an AUC of 1.000 (Figure 7F). However, given the very small sample size n=10, this estimate is potentially unstable and likely overoptimistic—even a single misclassification would substantially alter the AUC. This result is reported for completeness but should be interpreted with caution. The frozen nomogram achieved an AUC of 0.771 (95% CI: 0.62–0.92), with sensitivity 68.2% and specificity 71.4% at the Youden-optimal cutoff (Figure 7G), indicating modest but consistent discriminative performance in an independent cohort.

### 2.7. GSEA Enrichment Analysis of Hub PCD-DEGs

**Figure 8:**
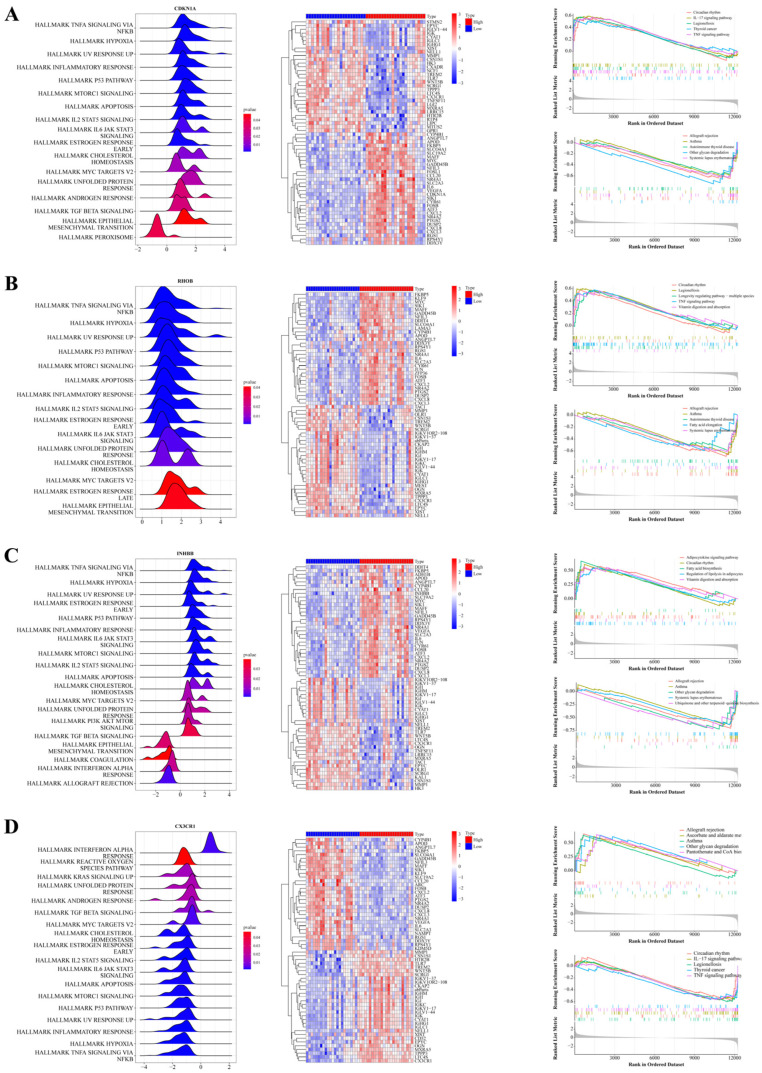
GSEA enrichment analysis of Hub PCD-DEG: (**A**–**D**) CDKN1A, RHOB, INHBB, and CX3CR1 were each subjected to GSEA HALLMARK pathway enrichment ridge mapping, differential expression heatmapping of high and low expression groups, and single-gene GSEA, respectively.

Hallmark pathway analysis revealed that CDKN1A, CX3CR1, INHBB, and RHOB were commonly enriched in TNFα/NF-κB, hypoxia, and UV response pathways (Figure 8A–D). CDKN1A, INHBB, and RHOB additionally co-enriched in p53 and apoptosis pathways, whereas CX3CR1 showed inverse enrichment. In high-expression cohorts, all four genes upregulated stress-response mediators (FKBP5, GADD45B) and inflammatory factors (IL6, CXCL8), and downregulated immune-modulatory genes (XIST, NELL1) and metabolic regulators (CYP4B1, APOD). Upregulated CDKN1A and RHOB enriched TNF signaling and stress pathways, while downregulated CX3CR1, INHBB, and RHOB suppressed autoimmune pathways. CX3CR1 downregulation uniquely attenuated ascorbate/aldarate metabolism, and RHOB upregulation enriched vitamin digestion pathways, indicating both convergent and gene-specific roles.

### 2.8. Predicting miRNA and TF of Hub PCD-DEGs

**Figure 9:**
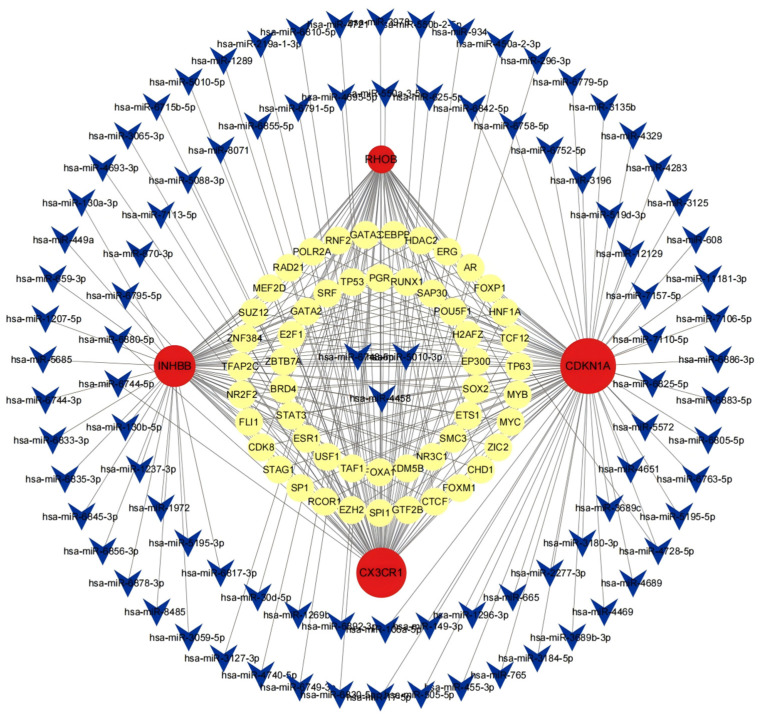
miRNA-TF-mRNA regulatory network of Hub PCD-DEG. Red circles represent genes; blue V shapes represent predicted miRNAs; yellow circles represent TFs.

We predicted the miRNAs and TFs that Hub PCD-DEGs might act on using an online database to visualize the regulatory network using Cytoscape. The network contains 151 nodes involving 306 edges, 235 miRNAs, and 54 TFs. hsa-miR-6748-5P, hsa-miR-5010-3p, and hsa-miR-4458 may be the acting target miRNAs coregulated by the Hub PCD-DEGs (Figure 9).

### 2.9. Immune Characteristics of OA

**Figure 10:**
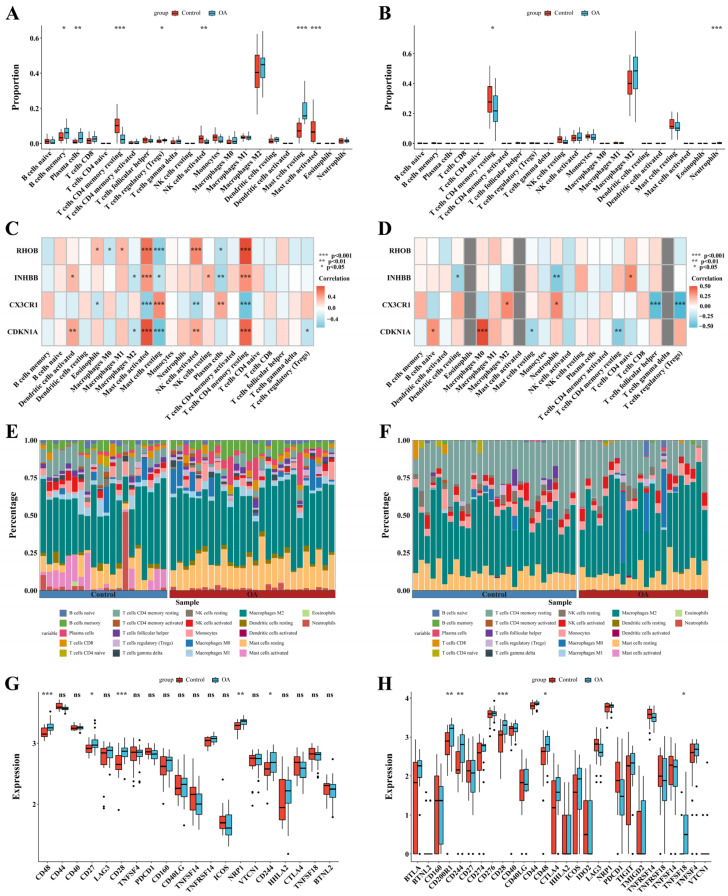
Immune infiltration analysis. (**A**,**B**) Box plots of differences in the infiltration of 22 immune cells in training sets and the GSE89408 dataset. (**C**,**D**) Correlation analysis of Hub PCD-DEGs with 22 immune cells in training sets and the GSE89408 dataset, * indicates *p* < 0.05, ** indicates *p* < 0.01, *** indicates *p* < 0.001. (**E**,**F**) The composition of 22 immune cells in each sample was plotted as a histogram in training sets and the GSE89408 dataset. (**G**,**H**) The expression differences in each chemokine gene in training sets and the GSE89408 dataset.

ssGSEA of the training set and GSE89408 dataset revealed that OA synovial samples had increased infiltration of memory B cells, plasma cells, Tregs, resting mast cells, and neutrophils, whereas CD4^+^ memory resting T cells, activated NK cells, and activated mast cells were decreased (Figure 10A,B). Spearman correlation analysis showed positive associations of RHOB, INHBB, and CDKN1A with activated mast cells, resting NK cells, and CD4^+^ memory resting T cells, and a negative association of CX3CR1 with these cells (*p <* 0.01 or *p <* 0.001) (Figure 10C,D). Immune cell composition was similar between datasets, with CD4^+^ memory resting T cells and M2 macrophages being most abundant (Figure 10E,F). Chemokine analysis identified CD48, CD27, CD28, NRP1, and CD244 as differentially expressed in the training set, and CD200R1, CD244, CD28, CD44, and TNFSF18 in GSE89408; CD244 and CD28 were consistently altered in both datasets (Figure 10G,H).

### 2.10. MDD Data Processing and Differential Genes Screening

**Figure 11:**
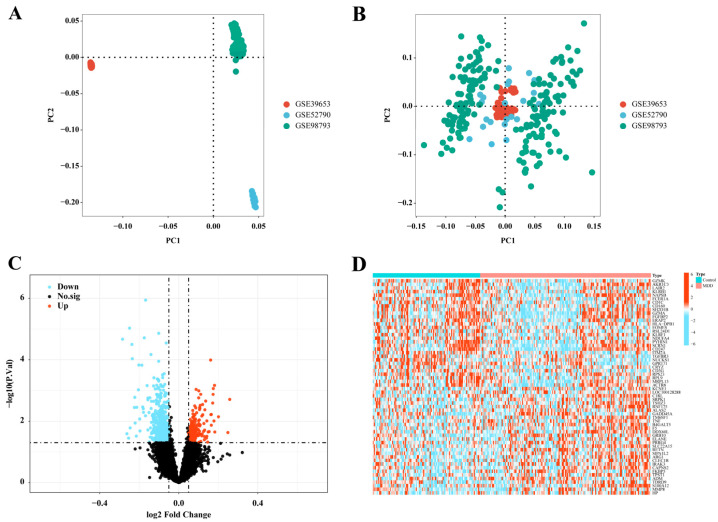
Data processing and DEGs analysis of MDD: (**A**,**B**) PCA plots demonstrating sample distribution before and after batch effect correction; (**C**) volcano plot of MDD DEGs; (**D**) heatmap of top 60 MDD DEGs showing expression patterns in normal samples vs. MDD samples.

Three MDD datasets from GEO were merged into a training set comprising 259 samples (100 normal, 159 MDD). UMAP analysis confirmed effective batch effect removal (Figure 11A,B). Using the limma R package, 723 DEGs were identified, including 219 upregulated and 504 downregulated genes in MDD (Table S6). The top 60 DEGs are shown in a volcano plot (Figure 11C) and a heatmap (Figure 11D).

### 2.11. WGCNA of MDD

**Figure 12:**
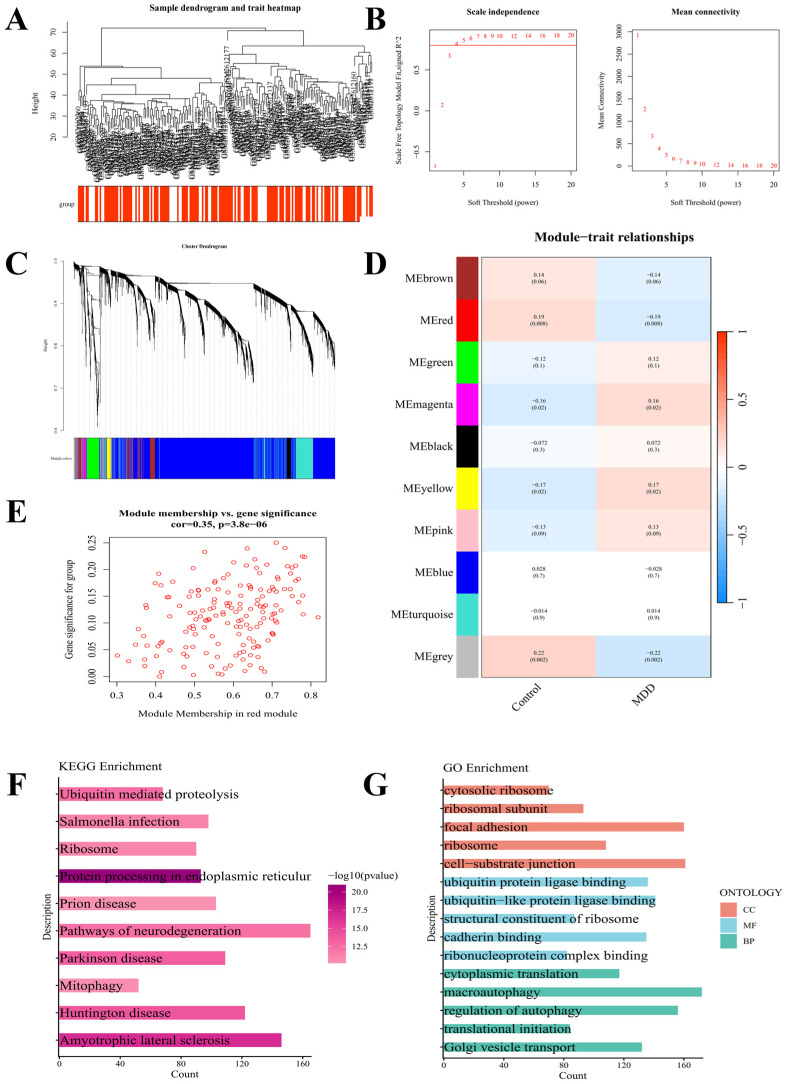
WGCNA of MDD. (**A**) Sample dendrogram and trait heatmap. (**B**) Determine the optimal soft threshold and construct a scale-free network by choosing nine based on where the red line (R^2^ = 0.9) is located. (**C**) The gene cluster dendrogram. (**D**) Heat map of module-trait relationships. (**E**) Scatter plot of correlations between gene significance (GS) and module membership (MM) in the red module. (**F**) KEGG enrichment of crucial genes within the red module. (**G**) GO enrichment of critical genes within the red module.

WGCNA was performed on the GSE98793 dataset to identify MDD-related gene modules. The soft-thresholding diagnostic analysis revealed that a power of β=4 was the optimal threshold where the topology fit index first established scale independence $\left(R^{2}>0.80\right)$ while preserving high mean connectivity. Unsupervised blockwise module detection clusterings partitioned the highly variable transcripts into 10 distinct co-expression modules. Three modules (yellow, red, and magenta), comprising 459 genes, showed significant correlations with MDD, with the red module exhibiting the strongest association (cor = 0.35, *p =* 3.8 × 10^−6^) (Figure 12A–E). Similarly, we conducted WGCNA analyses on two additional MDD training datasets—GSE653 and GSE52790—and integrated all significant modules (excluding gray modules) from the three datasets, identifying a total of 4467 genes associated with MDD. These genes were selected as hub genes (Table S3, S4 and S7). KEGG enrichment analysis indicated significant enrichment in protein processing in the endoplasmic reticulum, ubiquitin-mediated proteolysis, autophagy, phagosome, apoptosis, and pathways of neurodegeneration (Figure 12F). GO analysis revealed enrichment in biological processes, including cytoplasmic translation, macroautophagy, regulation of autophagy, RNA splicing, ribonucleoprotein complex biogenesis, and response to endoplasmic reticulum stress; cellular components, such as cytosolic ribosome, ribosomal subunit, spliceosomal complex, and focal adhesion; and molecular functions, including translation factor activity, RNA binding, and helicase activity (Figure 12G).

### 2.12. The Hub Co-Morbidity Genes Immune Characteristics of MDD

**Figure 13:**
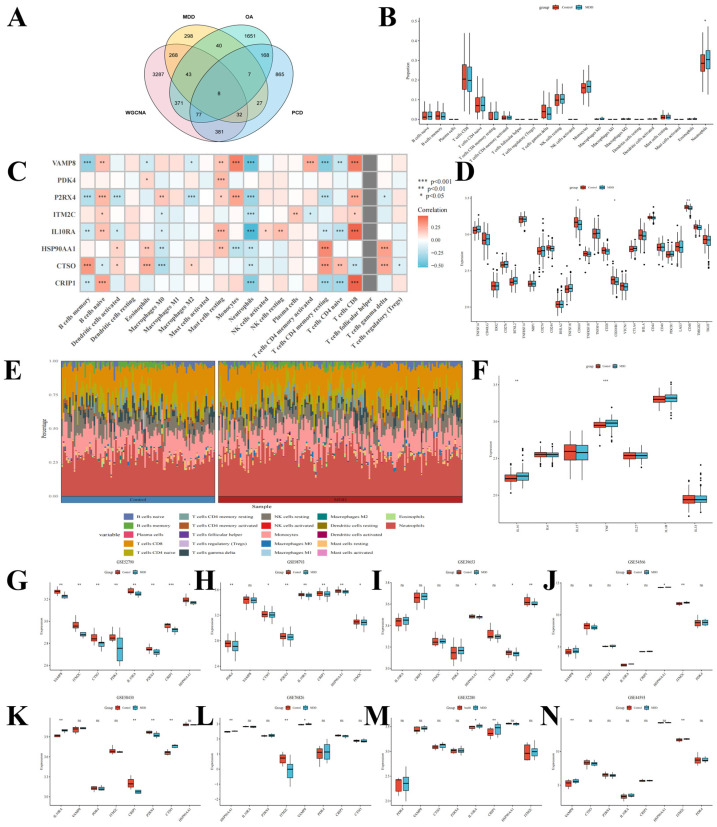
Identification and confirmation of key co-morbidity genes, along with analysis of immune infiltration. (**A**) Venn diagram of the hub co-morbidity genes screen. (**B**) Box plots of differences in the infiltration of 22 immune cells in MDD. (**C**) Correlation analysis of Hub Co-Morbidity genes with 22 immune cells in MDD. (**D**) Immune checkpoint analysis of Hub co-morbidity genes. (**E**) The composition of 22 immune cells in each sample was plotted as a histogram in MDD. (**F**) The expression differences in each chemokine gene in the training sets. (**G**–**N**) The hub co-morbidity genes were validated in eight datasets: GSE52790, GSE98793, GSE54566, GSE58430, GSE76826, GSE32280, and GSE44593. * *p <* 0.05, ** *p <* 0.01, *** *p <* 0.001.

Intersection of MDD, OA, PCD, and WGCNA datasets identified eight hub comorbidity genes: VAMP8, PDK4, P2RX4, ITM2C, IL10RA, HSP90AA1, CTSO, and CRIP1 (Figure 13A). Immune infiltration analysis showed significantly upregulated neutrophils in MDD (Figure 13B). These hub genes positively correlated with naive B cells and CD8^+^ T cells, and negatively correlated with memory B cells, M0 macrophages, and neutrophils (Figure 13C). Immune checkpoint analysis revealed differential expression of CD160, CD200R1, and CD48 (Figure 13D). MDD patients exhibited widespread alterations in peripheral immune cell profiles, including lymphocytes, innate immune cells, and chemokines IL-10 and TNF (Figure 13E,F). The hub genes were effectively validated across eight independent datasets (Figure 13G–N).

### 2.13. Single-Cell RNA Sequencing Analysis of the OA and MDD

**Figure 14:**
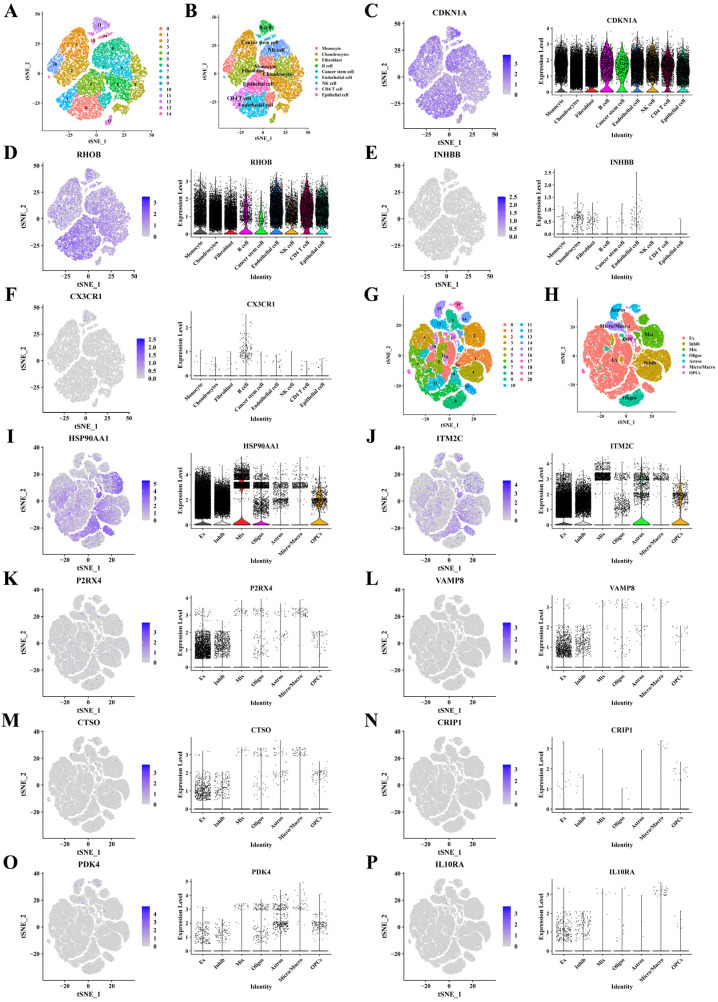
Single-cell RNA sequencing analysis of the OA synovium. (**A**,**B**) Cells in the GSE152805 dataset were clustered into 14 types through the t-SNE dimensionality reduction algorithm. (**C**–**F**) Histograms of features and scatter plots display the distribution of Hub PCD-DEGs across different cell types in the GSE152805 dataset. (**G**,**H**) Cells in the GSE144136 dataset were clustered into 21 types via the t-SNE dimensionality reduction algorithm. (**I**–**P**) Feature and scatter histograms showing the distribution of Hub Co-morbidity Genes across different cell types in the GSE144136 dataset.

Following the implementation of quality control procedures on the GSE152805 single-cell RNA dataset, PCA identified 15 distinct cellular subpopulations, including endothelial cells, fibroblasts, and various cell types such as endothelial cells, NK cells, monocytes, CD4 T cells, B cells, cancer stem cells, and endothelial cells (Figure 14A–C). It is noteworthy that we identified differential expression of hub PCD-DEGs among distinct cell clusters within the OA synovium (Figure 14D). Furthermore, CDKN1A and RHOB genes exhibited high levels of expression across various cell clusters. Conversely, INHBB and CX3CR1 exhibited minimal expression across all cell types in OA (Figure 14E,F). To gain a clearer understanding of cell cluster distribution in MDD and the expression differences in Hub PCD-DEGs, single-cell RNA analysis was applied to the GSE144136 dataset. Following a thorough quality control process on the GSE144136 dataset, principal component analysis (PCA) successfully identified 15 distinct cellular subpopulations. As demonstrated in Figure 14G,K, the following were observed: EX, Inhib, Mix, Oligos, Micro/Macro, Astros, and OPCs. Furthermore, high expression of the HSP90AA1 and ITM2C genes was detected in multiple cell clusters. In contrast, VAMP8, PDK4, P2RX4,1 LIORA, CTSO, and CRIP1 exhibited minimal expression in all cell types of MDD (Figure 14I–P).

### 2.14. Validation of Hub Genes at the Transcriptional and Protein Levels in Blood Samples from Patients with OA Complicated with MDD

**Figure 15:**
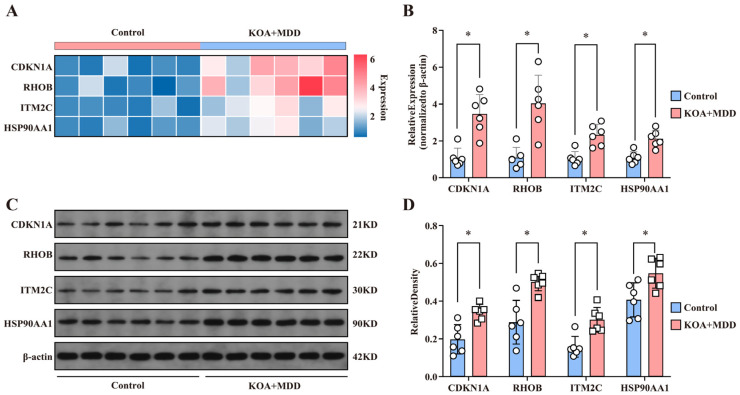
Experimental validation of hub genes in blood samples from normal controls and patients with OA–MDD using qRT-PCR and Western blot assays. (**A**) Heatmap showing mRNA expression levels of CDKN1A, RHOB, ITM2C, and HSP90AA1 in control and OA–MDD groups. (**B**) Bar graph of relative mRNA expression normalized to β-actin, with statistical significance indicated (* *p* < 0.05). (**C**) Representative Western blot bands for CDKN1A, RHOB, ITM2C, HSP90AA1, and β-actin in control and OA–MDD groups. (**D**) Bar graph of relative protein intensity quantified from Western blot band densities (* *p* < 0.05).

qRT-PCR and Western blot were used to verify the expression of CDKN1A, RHOB, ITM2C, and HSP90AA1 in blood samples from exactly 6 independent biological subjects per group (N=6 for Control; N=6 for OA–MDD comorbidity group). To maintain strict data independence and prevent artificial data inflation, the technical triplicates performed for each qRT-PCR run were averaged per individual, ensuring that each biological subject was treated as a single independent statistical unit. Detailed demographic information and relevant statistical data for the cohort members used in the wet laboratory validation experiments are presented in Table S8. And this cohort is independent of the GEO Discovery dataset. At the transcriptional level, all four hub genes were significantly upregulated in the OA–MDD group (*p* < 0.05), with RHOB showing the most prominent increase (Mean Fold Change = 4.02, 95% CI: 2.89–5.15) (Figure 15A,B). Upregulation was also observed for CDKN1A (Mean Fold Change = 3.46, 95% CI: 2.25–4.67), ITM2C (Mean Fold Change = 2.34, 95% CI: 1.72–2.96), and HSP90AA1 (Mean Fold Change = 2.11, 95% CI: 1.51–2.71). At the protein level, the expression of these four genes was also significantly higher in the OA–MDD group than in the control group (*p* < 0.05), as demonstrated by relative density differences for CDKN1A (difference = 0.14, 95% CI: 0.06–0.22), RHOB (difference = 0.22, 95% CI: 0.09–0.35), ITM2C (difference = 0.16, 95% CI: 0.07–0.25), and HSP90AA1 (difference = 0.14, 95% CI: 0.03–0.25) (Figure 15C,D). These results support the localized candidate expression patterns of CDKN1A, RHOB, ITM2C, and HSP90AA1, which are abnormally overexpressed at both mRNA and protein levels in the peripheral blood of OA–MDD comorbidity patients. Crucially, while single-cell transcriptomic profiling underscores discrete cell-type localization (such as CDKN1A/RHOB in OA synovial cell populations and HSP90AA1/ITM2C in MDD neuronal clusters) and clinical blood tissue evaluations demonstrate corresponding dysregulation, these observational findings do not prove a direct causal mechanism or demonstrate that these genes actively drive OA–MDD comorbidity pathogenesis. Rather, they highlight these molecules as prioritized candidate signatures that warrant functional validation in prospective experimental models.

## 3. Discussion

OA and MDD are leading causes of disability and mental health burden, with depression affecting up to 20% of OA patients [29]. Their shared inflammatory and oxidative stress pathways, particularly via programmed cell death (PCD), remain poorly understood [30]. Recent studies have increasingly highlighted that systemic inflammation, evidenced by elevated circulating cytokines and immune dysregulation, acts as a bridge between chronic musculoskeletal pain and neurobehavioral changes [31,32]. Our transcriptomic analysis identified 2365 DEGs in OA and 723 in MDD, including 24 PCD-DEGs in OA and eight hub comorbidity genes (VAMP8, PDK4, P2RX4, ITM2C, IL10RA, HSP90AA1, CTSO, CRIP1) common to both diseases. Enrichment analyses showed significant associations with immune–inflammatory pathways (TNF, PI3K-AKT, MAPK) in OA, and with apoptosis, cellular senescence, and neuroinflammation-related pathways in MDD, consistent with prior reports [33,34]. These findings are in line with previous bioinformatic reports that have independently identified TNF-mediated inflammatory cascades and apoptotic pathways as core pathogenic drivers in both OA synovitis and MDD-related neurodegeneration [35]. These findings suggest PCD pathways, especially apoptosis and pyroptosis, may be associated with OA–MDD comorbidity via shared stress-response mechanisms.

Using machine learning and ROC analyses (AUC > 0.9), we identified four hub PCD-DEGs (CDKN1A, CX3CR1, INHBB, RHOB) and eight comorbidity genes. CDKN1A has been linked to the regulation of apoptosis and senescence; its upregulation in OA may be associated with chondrocyte loss [36]. CX3CR1 has been reported to modulate immune recruitment; its downregulation correlates with suppressed autoimmunity [37]. INHBB (TGF-β family) is thought to support cartilage homeostasis [38], and RHOB has been linked to TNF signaling and apoptosis, which are associated with synovial inflammation [39]. Among comorbidity genes, HSP90AA1 and ITM2C were highly expressed in MDD neuronal clusters, potentially contributing to neuroinflammation and synaptic dysfunction [40]. Single-cell RNA-seq confirmed distinct expression patterns: CDKN1A and RHOB in OA synovium, HSP90AA1 and ITM2C in MDD. Immune infiltration revealed upregulated B cells, Tregs, and neutrophils in OA, and neutrophils in MDD, with significant correlations with hub genes (*p* < 0.01). These results highlight the diagnostic and therapeutic potential of these genes for integrated OA–MDD management. Further functional studies in animal models and clinical cohorts are needed to validate their precise roles.

Immune infiltration profiles differed between OA and MDD. OA showed increased memory B cells, plasma cells, Tregs, and neutrophils, while MDD featured neutrophil upregulation and altered lymphocyte subsets, consistent with prior reports [41]. Our observation of neutrophil activation aligns with recent meta-analyses demonstrating that elevated neutrophil-to-lymphocyte ratios (NLR) correlate with the severity of both inflammatory arthritis and depressive symptom burden [42]. Hub PCD-DEGs correlated with immune cells (e.g., positive correlations of RHOB, INHBB, CDKN1A with mast cells/T cells; negative correlation of CX3CR1). Comorbidity genes positively correlated with naive B cells and CD8^+^ T cells in MDD, and chemokines CD244/CD28 were differentially expressed in both diseases, supporting shared immune mechanisms [43].

WGCNA identified three MDD-associated modules; the gray module (cor = 0.22, *p =* 0.002) was most strongly associated. Its hub genes (VAMP8, PDK4, P2RX4, ITM2C, IL10RA, HSP90AA1, CTSO, CRIP1) were enriched in neuroinflammation-related pathways (protein processing, cellular senescence, apoptosis; *p* < 0.05) [44,45,46,47,48]. These genes were validated across eight datasets, supporting their diagnostic potential.

Single-cell RNA-seq revealed cell-type-specific expression: CDKN1A and RHOB were high in OA synovial cells; HSP90AA1 and ITM2C in MDD neuronal clusters. INHBB and CX3CR1 showed minimal expression in OA, highlighting context-dependent roles [49,50]. In blood samples from OA–MDD patients, qRT-PCR and Western blot confirmed significant upregulation of CDKN1A, RHOB, HSP90AA1, and ITM2C, corroborating the single-cell findings.

Several study limitations must be acknowledged. First, cross-tissue inference poses a challenge, as transcriptomic data from OA synovium and MDD brain tissues may introduce confounding tissue-specific effects. Second, this study lacks the baseline advantage of a dedicated, large-scale comorbid discovery cohort profiling both diseases simultaneously, relying instead on separate GEO datasets. Third, despite rigorous internal cross-validation, the high-dimensional machine-learning screening carries an inherent risk of possible ML overfitting and selection bias. Fourth, incomplete multiple-testing correction across certain multi-dataset integrations might introduce false positives. Fifth, the gray-module interpretation in WGCNA remains complex and less definitive due to the functional heterogeneity of unassigned transcripts. Finally, there is a lack of functional validation to prove whether these candidates actively drive or merely correlate with comorbidity pathogenesis. Future studies should address these gaps using larger prospective cohorts, rigorous mechanism-focused functional experiments, and multi-omics pipelines.

## 4. Materials and Methods

### 4.1. Data Collection and Processing

Gene expression datasets were acquired from the Gene Expression Omnibus (GEO) database, including 6 training datasets, 7 validation datasets, and 2 single-cell RNA sequencing datasets (Table 1). All preprocessing and batch effect removal were conducted exclusively within each training cohort. External validation datasets were not accessed, processed, or used in any way during training-phase operations. Three OA synovial tissue datasets and three MDD datasets were merged, background-corrected, and normalized via the limma R package(v3.62.1), followed by batch effect removal applied only within each training cohort using the ComBat empirical Bayes method in the SVA R package(v3.44.0) [51]. Batch correction parameters were computed only from training data. External validation datasets were processed independently using the same normalization procedure but were NOT included in the ComBat batch estimation step, ensuring no information leakage from validation to training data. Normalization parameters (scaling factors, model coefficients) were saved from the training cohort for independent re-application to external validation datasets. External validation was conducted using 2 independent OA synovial tissue samples and 5 MDD-related tissue samples, processed independently using the stored training-set parameters. The single-cell RNA sequencing datasets were used to characterize the pathological features of OA and MDD in subsequent analyses.

### 4.2. Download and Collate PCD-Related Genes

A comprehensive set of programmed cell death (PCD)-related genes was compiled by integrating data from FerrDb, HADb, and the Molecular Signatures Database (MSigDB). The collected genes covered diverse PCD modalities, including apoptosis, autophagy, necroptosis, pyroptosis, ferroptosis, and cuproptosis. After removing duplicates, 1986 non-redundant PCD-related genes were retained for subsequent analyses, and the full gene list is presented in Table S1.

### 4.3. Identification of DEGs in OA

Differential expression analysis between normal and OA synovial samples in the training set was performed using the limma R package [52], with differentially expressed genes (DEGs) defined by an absolute log2 fold change greater than 1 and a false discovery rate (FDR) below 0.05. Visualization of the results, including volcano plots and heatmaps, was carried out using the ggplot2 R package. The intersection of the identified DEGs with the compiled PCD-related gene set yielded the set of PCD-related DEGs (PCD-DEGs).

### 4.4. Functional Enrichment Analysis

Functional enrichment analyses were performed using the ClusterProfiler (v4.12.2) [53] and DOSE (v3.30.1) [54] R packages, with Kyoto Encyclopedia of Genes and Genomes (KEGG), gene ontology (GO), and disease ontology (DO) enrichment terms screened at a significance threshold of *p* < 0.05. Visualization of the enrichment results was conducted using the ggplot2 R package (v3.5.1) [55]. Additionally, cluster enrichment analysis of the PCD-DEGs, covering KEGG pathways and GO terms, was carried out using the Metascape database, with statistical significance also defined as *p* < 0.05.

### 4.5. GSVA Enrichment Analysis

Gene set variation analysis (GSVA), a non-parametric approach for evaluating pathway enrichment in transcriptomic data, was applied to characterize biological pathway activities [56]. Reference gene sets comprising “Hallmark.all.v2022.1.Hs.symbols” and “c2.cp.KEGG.symbols” were obtained from the Molecular Signatures Database (MSigDB) [57]. Enrichment scores for HALLMARK and KEGG pathways were calculated for normal and OA synovial samples using the GSVA R package, with statistical significance set at *p* < 0.05.

### 4.6. Protein–Protein Interaction Networks (PPI) and Correlation Analysis of PCD-DEGs

Protein–protein interaction (PPI) networks for PCD-DEGs were explored using the STRING database, with interactions retained at a confidence score threshold of 0.4. Network visualization was performed using Cytoscape (version 3.9.1), where nodes were ranked according to their degree scores [58]. Interactions among PCD-DEGs at the mRNA level were assessed using Pearson correlation analysis. Chromosomal localization information for these genes was retrieved from the ENSEMBL database and visualized using the RCircos R package [59].

### 4.7. Co-Expression Analysis of PCD-DEGs

The GeneMANIA database (http://genemania.org/) was employed to predict functionally related genes based on a comprehensive set of association data [60]. PCD-DEGs were submitted as input, with the analysis restricted to a maximum of 20 related genes and 10 functional attributes.

### 4.8. Identification of Hub PCD-DEGs

Three complementary machine learning algorithms—LASSO regression, SVM-RFE, and Random Forest—were applied to the OA training set only to screen hub PCD-DEGs. All feature selection and model tuning were conducted using a nested cross-validation framework, with external validation data held completely separate throughout. LASSO regression (glmnet R package) was implemented with a nested cross-validation structure: inner 10-fold cross-validation for λ optimization (minimum binomial deviance criterion), nested within an outer 5-fold cross-validation loop for unbiased AUC estimation [61]. This nested structure ensures that λ selection and performance evaluation use separate data partitions, preventing optimistic bias [62]. Final features were genes with non-zero coefficients at the optimal λ. SVM-RFE (e1071 R package) was implemented with embedded 5-fold recursive feature elimination. In each fold, features were iteratively removed; the subset achieving the maximum 5-fold cross-validation accuracy was selected [63]. Consensus features appearing across folds were retained to reduce fold-specific selection variance [64]. The Random Forest (randomForest R package) with a forest of 500 trees was grown with 10-fold cross-validation for variable importance ranking. Features with a mean decrease in Gini index > 1.0 were retained. Out-of-bag (OOB) error rates were monitored for consistency. The Venn diagram presents the hub PCD-DEGs, which are the PCD-related signature genes common to all three machine learning methods.

### 4.9. Construction of a Diagnostic Model for OA

A nomogram was constructed based on the expression levels of hub PCD-DEGs using the rms R package to enhance the clinical utility of OA diagnostic prediction [65]. Model fitting was performed exclusively on the OA training set. The predictive performance of the model was assessed through calibration curves, decision curves, and clinical impact curves. External validation was conducted on independent datasets GSE89408 and GSE1919 using the frozen nomogram coefficients without refitting or retuning.

### 4.10. ROC Curve Analysis and Differential Expression of Hub PCD-DEGs

The diagnostic performance of the hub PCD-DEGs and the constructed model was evaluated using receiver operating characteristic (ROC) analysis performed with the pROC R package [66]. Analyses were conducted on both the training set and the external validation datasets (GSE89408 and GSE1919). Differential expression levels of hub PCD-DEGs between normal and OA synovial samples were assessed using the Wilcoxon test.

### 4.11. Construction of the miRNA-TF-mRNA Regulatory Network of Hub PCD-DEGs

To predict miRNAs potentially regulating the hub PCD-DEGs, the miRDB [67] and miRTarBase databases [68] were utilized, and candidate miRNAs were retained by taking the intersection of predictions from these resources. We then used the hTFtarget [69], ENCODE [70], CHEA [71], GTRD [72] and KnockTF databases [73] to predict the transcription factors (TFs) that are potentially involved in the Hub PCD-DEGs. For screening, we used a *p*-value of 0.05 or less. The regulatory network that emerged was visualized in Cytoscape (version 3.9.1) with degree ranking.

### 4.12. Immune Infiltration Analysis

Immune infiltration analysis was performed using the CIBERSORT algorithm with the LM22 signature matrix to deconvolute bulk transcriptomic data and estimate the relative proportions of 22 immune cell subsets. Prior to analysis, the expression matrix underwent quantile normalization QN=TRUE, and 100 permutations perm=100 were conducted to evaluate the stability. Samples with a CIBERSORT *p*-value > 0.05 were excluded. The estimated proportions of the 22 immune cell types were subsequently extracted for downstream analyses. Differences in immune cell proportions between groups were compared using the Wilcoxon rank-sum test (validation cohort) or ANOVA (training cohort) and visualized with the ggpubr package. Correlations between immune cell proportions and key genes were assessed using the cor.test function and displayed as heatmaps generated with ggplot2. All statistical analyses were performed in R version 4.2.1, with a significance threshold of *p* < 0.05.

### 4.13. Gene Set Enrichment Analysis

Gene set enrichment analysis (GSEA) was applied to identify statistically significant differences in pathway activity between two biological states [74]. Samples in the training set were stratified into high- and low-expression groups based on the median expression level of each hub PCD-DEG. Enrichment analysis was performed using the “Hallmark.all.v2022.1.Hs.symbols” reference gene set from the MSigDB database to evaluate pathways and molecular mechanisms associated with differential expression of the hub PCD-DEGs between the two groups.

### 4.14. Identification of DEGs in MDD

The ConsensusClusterPlus R package was used for unsupervised clustering of MDD samples, based on DEG expression values with K set to 10. The ideal K value is found by evaluating the full cumulative distribution function (CDF) index. Principal component analysis (PCA) assesses the distribution of samples across clusters, and the Wilcox test evaluates DEG expression in MDD.

### 4.15. Molecular Characterization and Weighted Gene Co-Expression Network Analysis (WGCNA) in MDD

Gene set enrichment analysis (GSEA) was performed using GO, KEGG, and Hallmark gene sets from MSigDB to explore MDD-related biological functions. A weighted gene co-expression network was constructed with the WGCNA R package [75] using genes with absolute median expression difference > 0.25 in MDD samples. Genes were clustered into co-expression modules via the topological overlap matrix (TOM), and the module most significantly associated with MDD was selected. Hub genes in this module were identified at *p <* 0.05, followed by KEGG and GO enrichment analyses for functional annotation.

### 4.16. Screening of the Hub Co-Morbidity Genes and Immune Characteristics of MDD

Hub comorbidity genes were identified by intersecting the MDD, OA, WGCNA, and PCD datasets (Venn diagram). Single-sample gene set enrichment analysis (ssGSEA) was conducted with the GSVA R package [56] to calculate enrichment scores of immune cells and functions in normal and MDD samples [29]. The Wilcoxon test was used to assess the relative abundance of immune cells and chemokine expression, and the Spearman test was applied to analyze the correlations between hub comorbidity genes and immune cells/functions.

### 4.17. Single-Cell RNA Sequencing Analysis

Single-cell RNA-seq datasets (GSE152805 for OA synovium, GSE144136 for MDD) were analyzed using the Seurat R package. For the dataset, the Seurat object was created with min.features = 200 and min.cells = 3. Quality control was performed by filtering cells with nFeature_RNA > 200 and nFeature_RNA < 7000, and percent.mt < 15% (no ribosomal gene filtering was applied). No doublet detection strategy (e.g., DoubletFinder) was implemented. Data were normalized using the LogNormalize method with a scale.factor of 10,000, and 2000 highly variable genes were identified using the vst method. Principal component analysis was applied, followed by clustering with FindNeighbors dims=1:6 and FindClusters (resolution = 0.5). No batch effect integration via Harmony was performed (the relevant code was commented out). Differential expression genes were identified with min.pct = 0.25 and logfc.threshold = 0.25. Cell clusters were annotated using custom markers derived from the literature rather than the CellMarker database. Expression levels of hub PCD-DEGs and comorbidity genes were visualized across cell populations.

### 4.18. Study Subjects

This clinical validation study, combined with bioinformatics analysis, was approved by the Ethics Committee of the First Affiliated Hospital of Jiangxi University of Traditional Chinese Medicine (Approval No. JZFYLL20250103002). All participants provided written informed consent, and all procedures were conducted in compliance with relevant guidelines. A total of 60 subjects were recruited from the Outpatient Department of Acupuncture and Moxibustion of the hospital between June 2025 and May 2026. No significant differences in baseline characteristics (gender, age, disease duration) were found between groups (*p* > 0.05). OA was diagnosed according to the 2021 Diagnostic and Therapeutic Guidelines for Osteoarthritis of the Chinese Medical Association Orthopaedic Surgery Branch. MDD was diagnosed based on ICD-10 criteria with a HAMD-17 score ≥ 17. Inclusion criteria were age 18–80 years, suprapatellar bursa effusion ≥ 2 mm on knee ultrasound, and no systemic treatment for OA or MDD in the past 2 weeks. Exclusion criteria included pregnancy/lactation, long-term use of related drugs, severe knee joint deformity, and other major physical or mental comorbidities. Peripheral blood samples were collected from eligible subjects, cryopreserved, and used for subsequent molecular biological verification.

### 4.19. Experimental Validation

To verify the transcriptional and protein expression of hub genes (CDKN1A, RHOB, HSP90AA1, ITM2C) in the peripheral blood of OA patients, quantitative real-time PCR (qRT-PCR) and Western blot were conducted in this study. For qRT-PCR, total RNA was extracted using TRIzol reagent, and RNA purity was confirmed with an A260/A280 ratio of 1.8–2.0. RNA was reverse-transcribed into cDNA, and qPCR was performed on an ABI ViiA-7 system with SYBR Green. Primer sequences are listed in Table S2. The amplification procedure included pre-denaturation at 95 °C for 10 min, 40 cycles of denaturation at 95 °C for 10 s and annealing/extension at 60 °C for 60 s, followed by melting curve analysis. Relative gene expression was calculated using the 2^−ΔΔCt^ method with β-actin as the internal reference. For qRT-PCR, all samples were analyzed in technical triplicate to ensure procedural stability, and the triplicate values were averaged for each subject prior to statistical analysis. The final dataset utilized for both qRT-PCR and Western blot consisted of exactly 6 independent biological subjects per group (N=6 for Control; N=6 for KOA + MDD), ensuring that each subject was treated as an independent statistical unit. For Western blot, total protein was extracted with RIPA lysis buffer containing protease inhibitors, and protein concentration was determined by BCA assay. Equal protein was separated via 10% SDS-PAGE and transferred to PVDF membranes. After blocking with 5% non-fat milk, membranes were incubated with primary antibodies against target genes and β-actin at 4 °C overnight, then with HRP-conjugated secondary antibodies at room temperature for 2 h. Protein bands were visualized using ECL substrate, and band gray values were quantified with β-actin normalization.

### 4.20. Statistical Analysis

Statistical analyses were performed using R 4.2.2. Two-group comparisons were assessed using the Wilcoxon test. Quantitative real-time PCR data from at least three independent experiments are presented as mean ± SD. Statistical significance was determined by an unpaired two-tailed Student’s t-test, with significance levels denoted as * *p <* 0.05, ** *p <* 0.01, and *** *p <* 0.001.

## 5. Conclusions

In conclusion, our study highlights the critical roles of PCD-related and comorbidity genes in the inflammatory and immune dysregulation driving OA–MDD comorbidity. The identified biomarkers and immune profiles provide a foundation for developing integrated diagnostic and therapeutic strategies, potentially targeting shared pathways like TNF signaling and apoptosis to improve patient outcomes. Further experimental and clinical investigations are essential to translate these findings into precision medicine approaches.

## Figures and Tables

**Table 1:** All GEO datasets used in this study.

GEO (ID)	Platform	Tissue	Samples (Number)			Attribute
			Normal	OA	MDD	
GSE55235	GPL96	Synovial	10	10	0	Training (Ctrl vs. OA)
GSE55457	GPL96	Synovial	10	10	0	Training (Ctrl vs. OA)
GSE55584	GPL96	Synovial	0	6	0	Training (Ctrl vs. OA)
GSE39653	GPL10558	PBMC	24	0	21	Training (Ctrl vs. MDD)
GSE52790	GPL17976	PBMC	12	0	10	Training (Ctrl vs. MDD)
GSE98793	GPL570	whole blood	64	0	64	Training (Ctrl vs. MDD)
GSE1919	GPL91	Synovial	5	5	0	Validation (Ctrl vs. OA)
GSE89408	GPL11154	Synovial	28	22	0	Validation (Ctrl vs. OA)
GSE32280	GPL570	PBMC	8	0	15	Validation (Ctrl vs. MDD)
GSE44593	GPL570	AMY	14	0	14	Validation (Ctrl vs. MDD)
GSE54566	GPL570	AMY	14	0	14	Validation (Ctrl vs. MDD)
GSE58430	GPL14500	PBMC	6	0	6	Validation (Ctrl vs. MDD)
GSE76826	GPL17077	PBMC	12	0	10	Validation (Ctrl vs. MDD)
GSE152805	GPL20301	Synovial	0	3	0	Single cell (Hub gene localization)
GSE144136	GPL20301	DLPFC	17	0	17	Single cell (Hub gene localization)

Notes: DLPFC: dorsolateral prefrontal cortex; AMY: amygdala; DLPFC: dorsolateral prefrontal cortex; PBMC: peripheral blood mononuclear cells; GPL96 (Affymetrix Human Genome U133A Array); GPL10558 (Illumina HumanHT-12 V4.0 expression beadchip); GPL17976 (Affymetrix Human hGlue_3_0_v1 Array); GPL570 (Affymetrix Human Genome U133 Plus 2.0 Array); GPL91 (Affymetrix Human Genome U95A Array); GPL11154 (Illumina HiSeq 2000); GPL14550 (Agilent-028004 SurePrint G3 Human GE 8x60K Microarray); GPL17077 (Agilent-039494 SurePrint G3 Human GE v2 8x60K Microarray 039381); GPL20301 (Illumina HiSeq 4000). In the above list, all control groups in the OA dataset represent non-healthy surgical controls and non-healthy population controls.

## Data Availability

The datasets presented in this study are available in online repositories. The names of the repository/repositories and accession number(s) are provided in the article/Supplementary Material. Additional data supporting the conclusions of this study will be made available by the authors upon reasonable request.

## Supplementary Materials

The following supporting information can be downloaded at: https://www.mdpi.com/article/10.3390/ijms27125154/s1.

## Abbreviations

The following abbreviations are used in this manuscript:

OA	Osteoarthritis
MDD	Major depressive disorder
PCD	Programmed cell death
GEO	Gene Expression Omnibus
DEGs	Differentially expressed genes
WGCNA	Weighted gene co-expression network analysis
KEGG	Kyoto Encyclopedia of Genes and Genomes
GO	Gene ontology
DO	Disease ontology
MSigDB	Molecular Signatures Database
PPI	Protein–protein interaction networks
RFE	Recursive feature elimination
ROC	Receiver operating characteristic
TFs	Transcription factors
GSEA	Gene set enrichment analysis
CDF	Cumulative distribution function
PCA	Principal component analysis
ssGSEA	Single-sample gene set enrichment analysis

## References

[1] Zhang F., Rao S., Baranova A. (2022). Shared genetic liability between major depressive disorder and osteoarthritis. Bone Jt. Res..

[2] Barowsky S., Jung J.Y., Nesbit N., Silberstein M., Fava M., Loggia M.L., Smoller J.W., Lee P.H. (2021). Cross-Disorder Genomics Data Analysis Elucidates a Shared Genetic Basis Between Major Depression and Osteoarthritis Pain. Front. Genet..

[3] Yue S., Zhai G., Zhao S., Liang X., Liu Y., Zheng J., Chen X., Dong Y. (2024). The biphasic role of the infrapatellar fat pad in osteoarthritis. Biomed. Pharmacother..

[4] Mingoti M.E.D., Bertollo A.G., de Oliveira T., Ignácio Z.M. (2023). Stress and Kynurenine-Inflammation Pathway in Major Depressive Disorder. Adv. Exp. Med. Biol..

[5] Ruiz N.A.L., Del Ángel D.S., Brizuela N.O., Peraza A.V., Olguín H.J., Soto M.P., Guzmán D.C. (2022). Inflammatory Process and Immune System in Major Depressive Disorder. Int. J. Neuropsychopharmacol..

[6] Fransen M., Bridgett L., March L., Hoy D., Penserga E., Brooks P. (2011). The epidemiology of osteoarthritis in Asia. Int. J. Rheum. Dis..

[7] Litwic A., Edwards M.H., Dennison E.M., Cooper C. (2013). Epidemiology and burden of osteoarthritis. Br. Med. Bull..

[8] Claes S.J. (2009). Stress and depression: Clinical, neurobiological and genetical perspectives. Tijdschr. Psychiatr..

[9] Li H.Z., Liang X.Z., Sun Y.Q., Jia H.F., Li J.C., Li G. (2024). Global, regional, and national burdens of osteoarthritis from 1990 to 2021: Findings from the 2021 global burden of disease study. Front. Med..

[10] Yan G., Zhang Y., Wang S., Yan Y., Liu M., Tian M., Tian W. (2024). Global, regional, and national temporal trend in burden of major depressive disorder from 1990 to 2019: An analysis of the global burden of disease study. Psychiatry Res..

[11] Keshavarz K., Hedayati A., Rezaei M., Goudarzi Z., Moghimi E., Rezaee M., Lotfi F. (2022). Economic burden of major depressive disorder: A case study in Southern Iran. BMC Psychiatry.

[12] Leifer V.P., Katz J.N., Losina E. (2022). The burden of OA-health services and economics. Osteoarthr. Cartil..

[13] Zapata-Linares N., Berenbaum F., Houard X. (2024). Role of joint adipose tissues in osteoarthritis. Ann. Endocrinol..

[14] Mukherjee A., Das B. (2024). The role of inflammatory mediators and matrix metalloproteinases (MMPs) in the progression of osteoarthritis. Biomater. Biosyst..

[15] Nedunchezhiyan U., Varughese I., Sun A.R., Wu X., Crawford R., Prasadam I. (2022). Obesity, Inflammation, and Immune System in Osteoarthritis. Front. Immunol..

[16] Khalil M., Teunissen C.E., Otto M., Piehl F., Sormani M.P., Gattringer T., Barro C., Kappos L., Comabella M., Fazekas F. (2018). Neurofilaments as biomarkers in neurological disorders. Nat. Rev. Neurol..

[17] Maurya P.K., Noto C., Rizzo L.B., Rios A.C., Nunes S.O., Barbosa D.S., Sethi S., Zeni M., Mansur R.B., Maes M. (2016). The role of oxidative and nitrosative stress in accelerated aging and major depressive disorder. Prog. Neuropsychopharmacol. Biol. Psychiatry.

[18] Rawdin B.J., Mellon S.H., Dhabhar F.S., Epel E.S., Puterman E., Su Y., Burke H.M., Reus V.I., Rosser R., Hamilton S.P. (2013). Dysregulated relationship of inflammation and oxidative stress in major depression. Brain Behav. Immun..

[19] De Felici M., Piacentini M. (2015). Programmed Cell death in Development and Tumors. Int. J. Dev. Biol..

[20] Yao Q., Wu X., Tao C., Gong W., Chen M., Qu M., Zhong Y., He T., Chen S., Xiao G. (2023). Osteoarthritis: Pathogenic signaling pathways and therapeutic targets. Signal Transduct. Target. Ther..

[21] Kong P., Ahmad R.E., Zulkifli A., Krishnan S., Nam H.Y., Kamarul T. (2024). The role of autophagy in mitigating osteoarthritis progression via regulation of chondrocyte apoptosis: A review. Jt. Bone Spine.

[22] Lu Y., Zhou J., Wang H., Gao H., Ning E., Shao Z., Hao Y., Yang X. (2024). Endoplasmic reticulum stress-mediated apoptosis and autophagy in osteoarthritis: From molecular mechanisms to therapeutic applications. Cell Stress Chaperones.

[23] Liu K., Wang M., Li D., Duc Duong N.T., Liu Y., Ma J., Xin K., Zhou Z. (2024). PANoptosis in autoimmune diseases interplay between apoptosis, necrosis, and pyroptosis. Front. Immunol..

[24] Samir P., Malireddi R.K.S., Kanneganti T.D. (2020). The PANoptosome: A Deadly Protein Complex Driving Pyroptosis, Apoptosis, and Necroptosis (PANoptosis). Front. Cell. Infect. Microbiol..

[25] Xu H. (2025). Editorial for the Bioinformatics of Human Diseases Special Issue. Genes.

[26] Kumar A., Ahmad S.F., Mukhopadhyay C.S., Choudhary R.K., Panwar H., Malik Y.S. (2023). Bioinformatics: Unveiling the Systems Biology. Biotechnological Interventions Augmenting Livestock Health and Production.

[27] Katsoula G., Kreitmaier P., Zeggini E. (2022). Insights into the molecular landscape of osteoarthritis in human tissues. Curr. Opin. Rheumatol..

[28] Shukla R., Newton D.F., Sumitomo A., Zare H., McCullumsmith R., Lewis D.A., Tomoda T., Sibille E. (2022). Molecular characterization of depression trait and state. Mol. Psychiatry.

[29] Gong W., Kuang M., Chen H., Luo Y., You K., Zhang B., Liu Y. (2024). Single-sample gene set enrichment analysis reveals the clinical implications of immune-related genes in ovarian cancer. Front. Mol. Biosci..

[30] Juhasz G., Chase D., Pegg E., Downey D., Toth Z.G., Stones K., Platt H., Mekli K., Payton A., Elliott R. (2009). CNR1 gene is associated with high neuroticism and low agreeableness and interacts with recent negative life events to predict current depressive symptoms. Neuropsychopharmacology.

[31] Capuron L., Miller A.H. (2011). Immune system to brain signaling: Neuropsychopharmacological implications. Pharmacol. Ther..

[32] Miller A.H., Raison C.L. (2016). The role of inflammation in depression: From evolutionary imperative to modern treatment target. Nat. Rev. Immunol..

[33] Chen P., Zhou J., Ruan A., Guan H., Xie J., Zeng L., Liu J., Wang Q. (2022). Synovial tissue-derived extracellular vesicles induce chondrocyte inflammation and degradation via NF-κB signalling pathway: An in vitro study. J. Cell. Mol. Med..

[34] Chen J., Xie X., Lin M., Han H., Wang T., Lei Q., He R. (2024). Genes associated with cellular senescence as diagnostic markers of major depressive disorder and their correlations with immune infiltration. Front. Psychiatry.

[35] Liu T., Zhang L., Joo D., Sun S.C. (2017). NF-κB signaling in inflammation. Signal Transduct. Target. Ther..

[36] Fang C., Zhu S., Zhong R., Yu G., Lu S., Liu Z., Gao J., Yan C., Wang Y., Feng X. (2024). CDKN1A regulation on chondrogenic differentiation of human chondrocytes in osteoarthritis through single-cell and bulk sequencing analysis. Heliyon.

[37] Yano R., Yamamura M., Sunahori K., Takasugi K., Yamana J., Kawashima M., Makino H. (2007). Recruitment of CD16+ monocytes into synovial tissues is mediated by fractalkine and CX3CR1 in rheumatoid arthritis patients. Acta Medica Okayama.

[38] Thielen N.G.M., van der Kraan P.M., van Caam A.P.M. (2019). TGFβ/BMP Signaling Pathway in Cartilage Homeostasis. Cells.

[39] Kim D.M., Chung K.S., Choi S.J., Jung Y.J., Park S.K., Han G.H., Ha J.S., Song K.B., Choi N.S., Kim H.M. (2009). RhoB induces apoptosis via direct interaction with TNFAIP1 in HeLa cells. Int. J. Cancer.

[40] Yoshino Y., Roy B., Kumar N., Shahid Mukhtar M., Dwivedi Y. (2021). Molecular pathology associated with altered synaptic transcriptome in the dorsolateral prefrontal cortex of depressed subjects. Transl. Psychiatry.

[41] Mimpen J.Y., Hedley R., Ridley A., Baldwin M.J., Windell D., Bhalla A., Ramos-Mucci L., Buckley C.D., Coles M.C., Alvand A. (2023). Cellular characterisation of advanced osteoarthritis knee synovium. Arthritis Res. Ther..

[42] Chen S., Ying H., Du J., Zhu X., Shi J., Zhang Y., Chen S., Shen B., Li J. (2019). The association between albumin-dNLR score and disease activity in patients with rheumatoid arthritis. J. Clin. Lab. Anal..

[43] Sun L., Gang X., Li Z., Zhao X., Zhou T., Zhang S., Wang G. (2021). Advances in Understanding the Roles of CD244 (SLAMF4) in Immune Regulation and Associated Diseases. Front. Immunol..

[44] Torres-Odio S., Key J., Hoepken H.H., Canet-Pons J., Valek L., Roller B., Walter M., Morales-Gordo B., Meierhofer D., Harter P.N. (2017). Progression of pathology in PINK1-deficient mouse brain from splicing via ubiquitination, ER stress, and mitophagy changes to neuroinflammation. J. Neuroinflammation.

[45] Sepehrinezhad A., Bozorgmehr A., Negah S.S., Karimi M., Shahbazi A. (2020). Neuroinflammation and Disrupted Synaptic Plasticity, the Main Pathological Processes in Multiple Sclerosis and Obsessive-Compulsive Disorder: An Enrichment Analysis. Res. Sq..

[46] Lv W., Jiang J., Xu Y., Chen Z., Wang Z., Xing A., Zheng X., Qu T., Wan Q. (2023). Re-Exploring the Inflammation-Related Core Genes and Modules in Cerebral Ischemia. Mol. Neurobiol..

[47] Li S., Lu C., Zhao Z., Lu D., Zheng G. (2023). Uncovering neuroinflammation-related modules and potential repurposing drugs for Alzheimer’s disease through multi-omics data integrative analysis. Front. Aging Neurosci..

[48] Pastrello C., Niu Y., Jurisica I. (2022). Pathway Enrichment Analysis of Microarray Data. Methods Mol. Biol..

[49] Hellwig S., Brioschi S., Dieni S., Frings L., Masuch A., Blank T., Biber K. (2016). Altered microglia morphology and higher resilience to stress-induced depression-like behavior in CX3CR1-deficient mice. Brain Behav. Immun..

[50] Zhang H., Wang Z., Zhou Q., Cao Z., Jiang Y., Xu M., Liu J., Zhou J., Yan G., Sun H. (2023). Downregulated INHBB in endometrial tissue of recurrent implantation failure patients impeded decidualization through the ADCY1/cAMP signalling pathway. J. Assist. Reprod. Genet..

[51] Jeffrey T.L., Johnson W.E., Hilary S.P., Andrew E.J., John D.S. (2012). The sva package for removing batch effects and other unwanted variation in high-throughput experiments. Bioinformatics.

[52] Ritchie M.E., Phipson B., Wu D., Hu Y., Law C.W., Shi W., Smyth G.K. (2015). limma powers differential expression analyses for RNA-sequencing and microarray studies. Nucleic Acids Res..

[53] Yu G., Wang L.G., Han Y., He Q.Y. (2012). clusterProfiler: An R package for comparing biological themes among gene clusters. Omics.

[54] Ritz C., Baty F., Streibig J.C., Gerhard D. (2015). Dose-Response Analysis Using R. PLoS ONE.

[55] Gustavsson E.K., Zhang D., Reynolds R.H., Garcia-Ruiz S., Ryten M. (2022). ggtranscript: An R package for the visualization and interpretation of transcript isoforms using ggplot2. Bioinformatics.

[56] Hänzelmann S., Castelo R., Guinney J. (2013). GSVA: Gene set variation analysis for microarray and RNA-seq data. BMC Bioinform..

[57] Castanza A.S., Recla J.M., Eby D., Thorvaldsdóttir H., Bult C.J., Mesirov J.P. (2023). Extending support for mouse data in the Molecular Signatures Database (MSigDB). Nat. Methods.

[58] Szklarczyk D., Kirsch R., Koutrouli M., Nastou K., Mehryary F., Hachilif R., Gable A.L., Fang T., Doncheva N.T., Pyysalo S. (2023). The STRING database in 2023: Protein-protein association networks and functional enrichment analyses for any sequenced genome of interest. Nucleic Acids Res..

[59] Zhang H., Meltzer P., Davis S. (2013). RCircos: An R package for Circos 2D track plots. BMC Bioinform..

[60] Zuberi K., Franz M., Rodriguez H., Montojo J., Lopes C.T., Bader G.D., Morris Q. (2013). GeneMANIA prediction server 2013 update. Nucleic Acids Res..

[61] Xi L.J., Guo Z.Y., Yang X.K., Ping Z.G. (2023). Application of LASSO and its extended method in variable selection of regression analysis. Zhonghua Yu Fang Yi Xue Za Zhi.

[62] Huang A., Liu D. (2021). EBglmnet: A comprehensive R package for sparse generalized linear regression models. Bioinformatics.

[63] Rahman N.A.A., Nasarudin N.A., Mohamad M.S. (2024). Pathway-Based Analysis Using SVM-RFE for Gene Selection and Classification. AI Technologies and Virtual Reality.

[64] Todorov V. (2024). The R Package Ecosystem for Robust Statistics. WIREs Comput. Stat..

[65] Clarke W.T., Stagg C.J., Jbabdi S. (2021). FSL-MRS: An end-to-end spectroscopy analysis package. Magn. Reson. Med..

[66] Robin X., Turck N., Hainard A., Tiberti N., Lisacek F., Sanchez J.C., Müller M. (2011). pROC: An open-source package for R and S+ to analyze and compare ROC curves. BMC Bioinform..

[67] Yang J.H., Li J.H., Shao P., Zhou H., Chen Y.Q., Qu L.H. (2011). starBase: A database for exploring microRNA-mRNA interaction maps from Argonaute CLIP-Seq and Degradome-Seq data. Nucleic Acids Res..

[68] Chou C.H., Shrestha S., Yang C.D., Chang N.W., Lin Y.L., Liao K.W., Huang W.C., Sun T.H., Tu S.J., Lee W.H. (2018). miRTarBase update 2018: A resource for experimentally validated microRNA-target interactions. Nucleic Acids Res..

[69] Zhang Q., Liu W., Zhang H.M., Xie G.Y., Miao Y.R., Xia M., Guo A.Y. (2020). hTFtarget: A Comprehensive Database for Regulations of Human Transcription Factors and Their Targets. Genom. Proteom. Bioinform..

[70] Hitz B.C., Jin-Wook L., Jolanki O., Kagda M.S., Graham K., Sud P., Gabdank I., Strattan J.S., Sloan C.A., Dreszer T. (2023). The ENCODE Uniform Analysis Pipelines. bioRxiv.

[71] Lachmann A., Xu H., Krishnan J., Berger S.I., Mazloom A.R., Ma’ayan A. (2010). ChEA: Transcription factor regulation inferred from integrating genome-wide ChIP-X experiments. Bioinformatics.

[72] Kolmykov S., Yevshin I., Kulyashov M., Sharipov R., Kondrakhin Y., Makeev V.J., Kulakovskiy I.V., Kel A., Kolpakov F. (2021). GTRD: An integrated view of transcription regulation. Nucleic Acids Res..

[73] Feng C., Song C., Liu Y., Qian F., Gao Y., Ning Z., Wang Q., Jiang Y., Li Y., Li M. (2020). KnockTF: A comprehensive human gene expression profile database with knockdown/knockout of transcription factors. Nucleic Acids Res..

[74] Zito A., Lualdi M., Granata P., Cocciadiferro D., Novelli A., Alberio T., Casalone R., Fasano M. (2021). Gene Set Enrichment Analysis of Interaction Networks Weighted by Node Centrality. Front. Genet..

[75] Liu Y. (2024). CWGCNA: An R package to perform causal inference from the WGCNA framework. NAR Genom. Bioinform..

