# Supplementary figures and images for: Integrated Bulk and Single-Cell Transcriptomic Analysis Followed by Clinical Validation Reveal Programmed Cell Death-Related Shared Molecular Signatures in OA and MDD
Source: Int J Mol Sci. 2026 Jun 6;27(12):5154. doi: 10.3390/ijms27125154 (PMC13300034; doi:10.3390/ijms27125154)

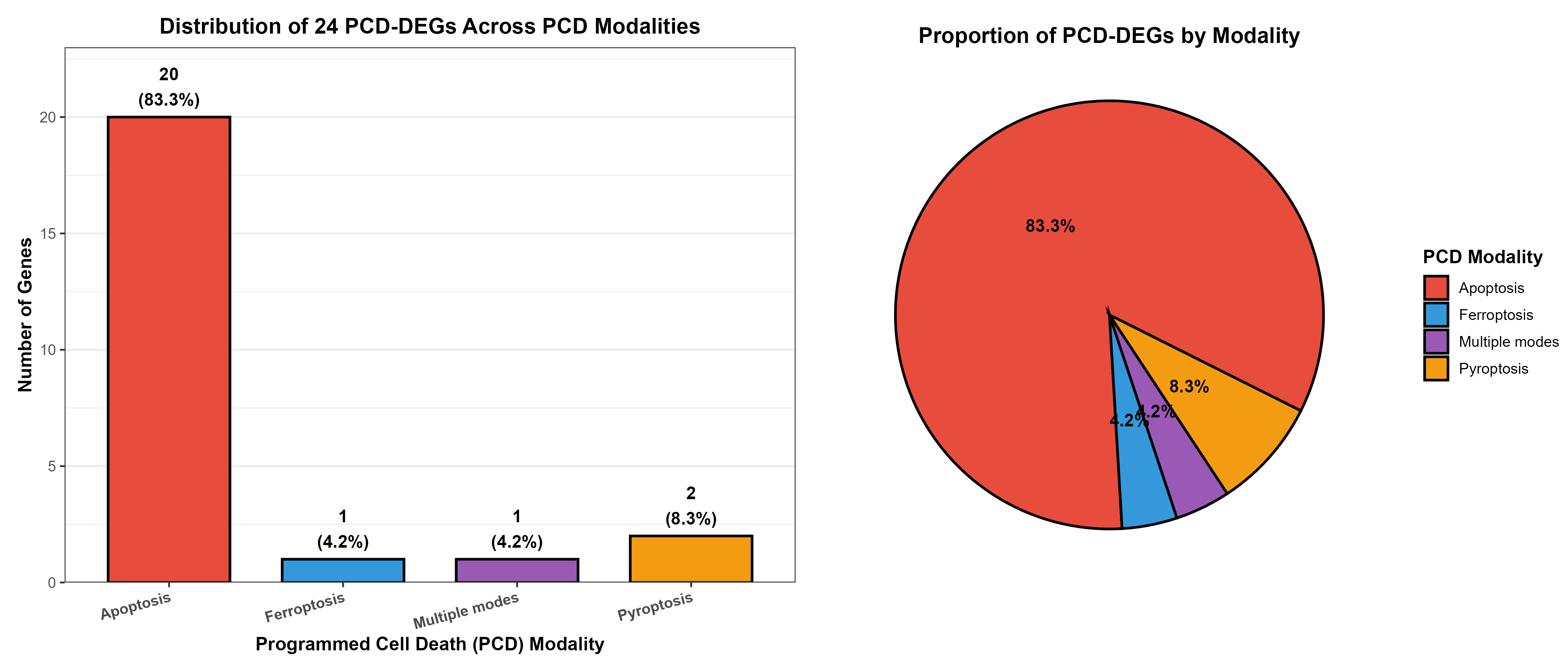

Supplement: Supplementary file 1 [file ijms-27-05154-s001.zip › Supplementary Figure S1.png]

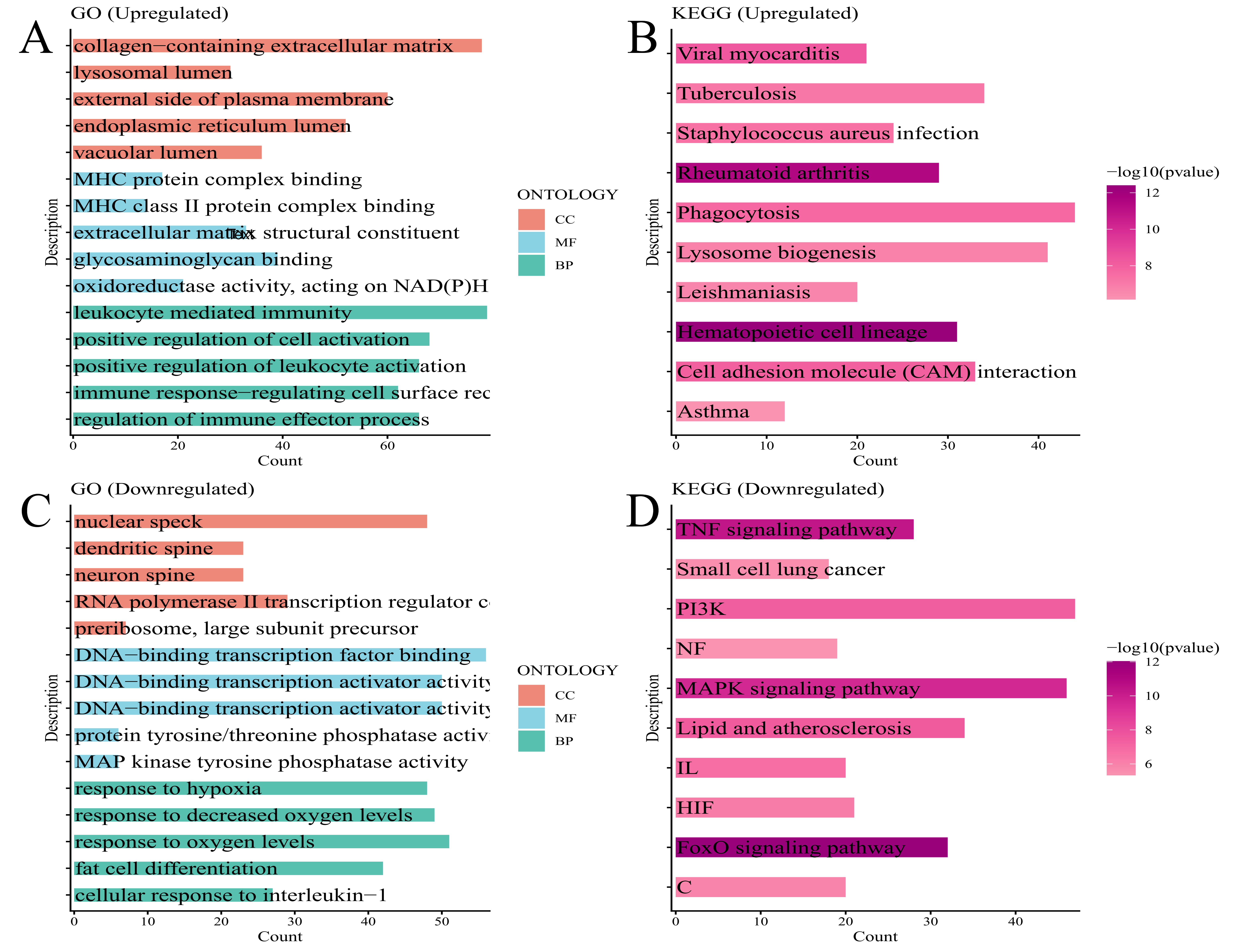

Supplement: Supplementary file 1 [file ijms-27-05154-s001.zip › Supplementary Figure S2.png]

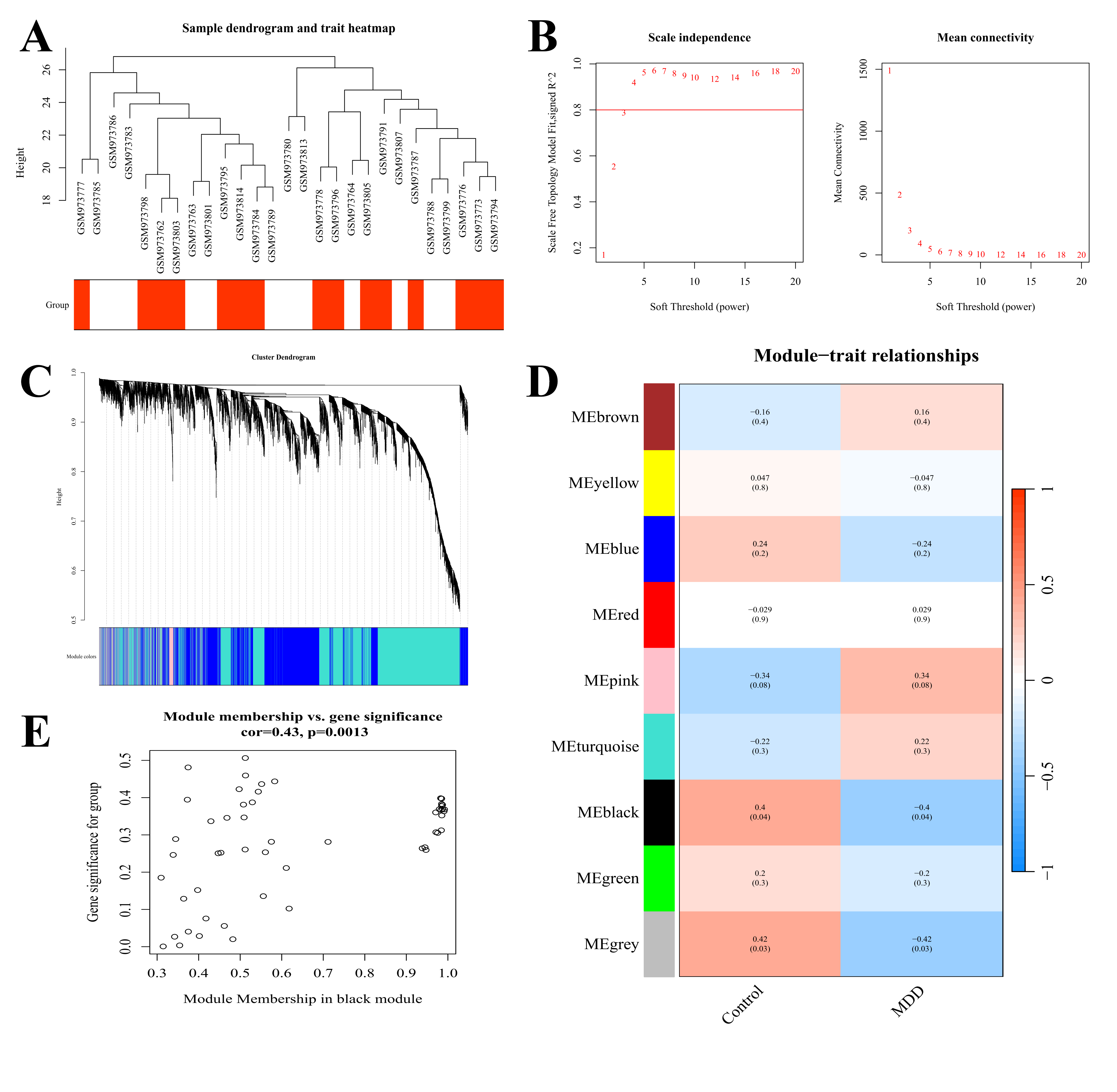

Supplement: Supplementary file 1 [file ijms-27-05154-s001.zip › Supplementary Figure S3.png]
